# Synthesis and Biological Studies on (KLAKLAK)_2_-NH_2_ Analog Containing Unnatural Amino Acid β-Ala and Conjugates with Second Pharmacophore

**DOI:** 10.3390/molecules26237321

**Published:** 2021-12-02

**Authors:** Sirine Jaber, Veronica Nemska, Ivan Iliev, Elena Ivanova, Tsvetelina Foteva, Nelly Georgieva, Ivan Givechev, Emilia Naydenova, Veronika Karadjova, Dancho Danalev

**Affiliations:** 1 University of Chemical Technology and Metallurgy, 8 Kliment Ohridski Blvd, 1756 Sofia, Bulgaria; Jaber-Sirine@hotmail.com (S.J.); revn@abv.bg (V.N.); tsvetelina_angelova@abv.bg (T.F.); neli@uctm.edu (N.G.); e_naydenova@abv.bg (E.N.); vkar@mail.bg (V.K.); 2 Institute of Experimental Morphology, Pathology and Anthropology with Museum, Bulgarian Academy of Sciences, Acad. G. Bonchev Str., bl. 25 A, 1113 Sofia, Bulgaria; taparsky@abv.bg (I.I.); elena9512@abv.bg (E.I.); 3 Testing Center Global Test Ltd., 31 Krushovski vrah Street, 1618 Sofia, Bulgaria; ivangivechev@gmail.com

**Keywords:** (KLAKLAK)_2_, antitumor peptides, 1,8-naphthalimide, caffeic acid, unnatural amino acids

## Abstract

(1) Background: Peptides are good candidates for anticancer drugs due to their natural existence in the body and lack of secondary effects. (KLAKLAK)_2_ is an antimicrobial peptide that also shows good anticancer properties. (2) Methods: The Solid Phase Peptide Synthesis (Fmoc-strategy) was used for the synthesis of target molecules, analogs of (KLAKLAK)_2_-NH_2_. The purity of all compounds was monitored by HPLC, and their structures were proven using mass spectrometry. Cytotoxicity and antiproliferative effects were studied using 3T3 NRU and MTT tests, respectively. For determination of antimicrobial activity, the disc-diffusion method was used. Hydrolytic stability at three pH values, which mimic the physiological pH in the body, was investigated by means of the HPLC technique. (3) Results: A good selective index against MCF-7 tumor cell lines, combined with good cytotoxicity and antiproliferative properties, was revealed for conjugates NphtG-(KLAKLAK)_2_-NH_2_ and Caf-(KLAKLAK)_2_-NH_2_. The same compounds showed very good antifungal properties and complete hydrolytic stability for 72 h. The compound Caf-(KLβ-AKLβ-AK)_2_-NH_2_ containing β-Ala in its structures exhibited good antimicrobial activity against *Escherichia coli* K12 407 and *Bacillus subtilis* 3562, in combination with very good antiproliferative and cytotoxic properties, as well as hydrolytic stability. (4) Conclusions: The obtained results reveal that all synthesized conjugates could be useful for medical practice as anticancer or antimicrobial agents.

## 1. Introduction

Currently, millions of people worldwide suffer from some kind of cancer. According to the data of the GLOBOCAN database, cancer diseases increased in 2020 to 19.3 million new cases, and by 2040, the database predicts more than 30 million [1]. Together with cardiovascular diseases, malignancies are the leading cause of mortality in the world. Unfortunately, traditional chemotherapeutics have many disadvantages, including lack of selectivity affecting health cells and undesired secondary effects such as nausea, vomiting and strong secondary effects throughout the whole body. Possible alternatives to conventional chemotherapeutics are different natural products with established anticancer properties [2,3] and molecules that can be associated with the normal function of the body as part of its metabolic processes such as peptides, carbohydrates, etc. The latter possess different biological activities such as antiviral [4,5], anticancer [6,7], analgesic [8,9], etc. [10]. They have the advantage that, as part of the structure and metabolic processes of the body, they do not show any secondary effects, or any undesired effects are relatively weak. The numerous peptides and peptide mimetics introduced into medical practice for the treatment of various diseases, such as antiplatelet [11], antiviral [12], etc., medical drugs, and in particular as antitumor agents [13], are evidence of this fact. Different mechanisms of action of peptides on the tumor cell have been revealed. One group of peptides, somatostatin [14] and its analogs [15,16], which are already used in medical practice, binds to specific receptors or inhibits hormones with a key role in tumor cell proliferation [17,18,19]. Another group of peptides, antimicrobial peptides, possess secondary anticancer activity due to their ability to penetrate the cell membrane of tumor cells and activate the apoptosis process [20,21]. A representative of the second group of peptides is the antimicrobial peptide (KLAKLAK)_2_ [22,23]. On internalization, (KLAKLAK)_2_ causes mitochondrial swelling and destruction of the mitochondrial membrane, leading to apoptosis [24]. Javadpour et al. described the synthesis of analogs of (KLAKLAK)_2_ as C-terminal amides, and their study revealed that the newly synthesized peptides were much less lytic toward human erythrocytes than 3T3 cells at concentrations lower than 22 μM [22]. Taking into account that the introduction of unnatural amino acids in the primary structure of peptides could lead to higher activity and stability, in our previous work, we reported the synthesis and some investigations of the anticancer ability of the monomer KLAKLAK-NH_2_ peptide containing amino acids β-Ala and nor-Leu (Nle) in its structure [25]. However, Javadpour et al. reported that a minimal (KLAKLAK)_2_ sequence is necessary for some activity. Our study reveals that the introduction of β-Ala instead of Ala in the single sequence leads to high biological activity and selectivity according to the tested cell lines. In addition, during the study, the synthesis of a hybrid structure with a second pharmacophore with good anticancer properties was also reported. In addition, a paper published by Cabrele and coworkers reveals many positive aspects of introducing β-amino acids to the primary structures of peptides, especially for those with medical applications. They described that a peptide containing such peptide moieties display various biological functions, including antimicrobial activity, inhibition of protein−protein interactions, agonism/antagonism of GPCR ligands and antiangiogenic activity [26]. Taking into account all the above, herein, we report the synthesis and biological studies of the doubled sequence (KLAKLAK)_2_-NH_2_, replacing in its primary structure Ala with β-Ala. Encouraged by the good biological activity of the shortened analogs of (KLAKLAK)_2_-NH_2_, hybrid structures with the second pharmacophore 1,8-naphthalimide (Npht) and caffeic acid (Caf) were also synthesized and investigated for their anticancer potential.

## 2. Results

### 2.1. Synthesis and Characterization of Target Compounds

**Scheme 1:**
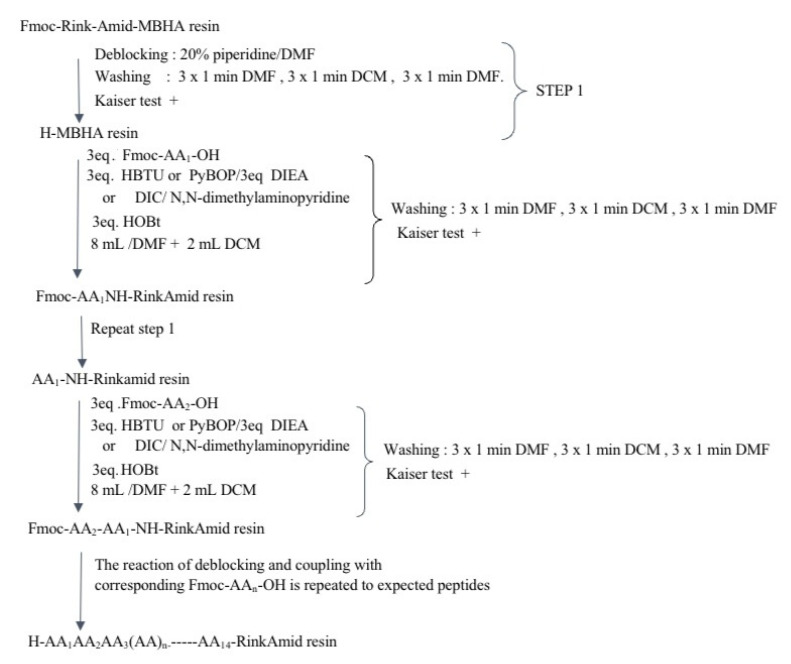
General scheme for synthesis of target compounds using SPPS.

A series of analogs of (KLAKLAK)_2_ were synthesized as C-terminal amides with the general structure (LysLeuXLysLeuXLys)_2_-NH_2_, where X is Ala or β-Ala. In addition, their conjugates with the main structure YLysLeuXLysLeuXLys-NH_2_, where X is Ala or β-Ala and Y is 1,8-naphthalimide-Gly (NphtG) or caffeic acid (Caf), were also obtained. All compounds were synthesized using the SPPS, Fmoc/Ot-Bu strategy according to Scheme 1.

The required C-terminal amides were obtained using Rink amide MBHA resin as a solid-phase carrier. All condensation steps were realized with HBTU, DIC or PyBOP. 1,8-Naphthalimideglycine was synthesized according to Marinov et al. [27]. Analytical data for newly synthesized peptides are summarized in Table 1.

### 2.2. Cytotoxicity

**Figure 1:**
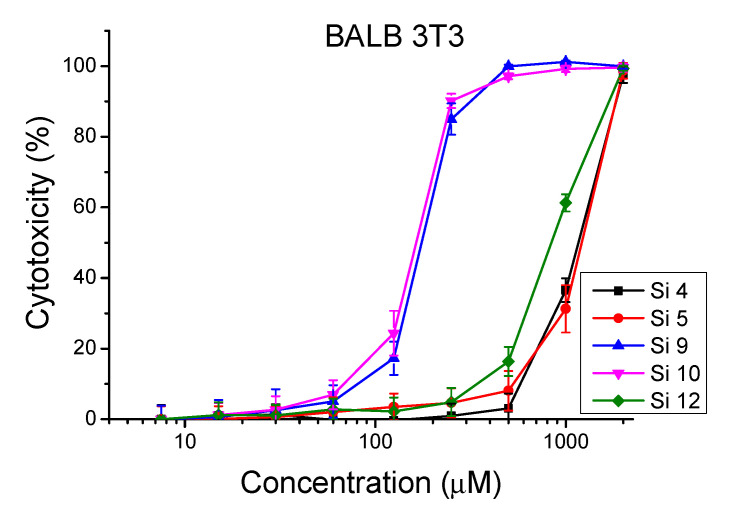
Cytotoxic effects of newly synthesized compounds. Dose–response curves for cytotoxicity assessment in BALB 3T3 cells. Values are means ± standard deviation from three independent experiments, *n* = 6.

The newly synthesized compounds were studied for cytotoxicity assessment by an in vitro 3T3 NRU test. The cells were incubated with the test substances at a concentration of 15 to 2000 μM for 24 h at 37 °C, 5% CO_2_ and 95% humidity. The cytotoxicity expressed in % relative to the negative control was determined. Dose–response dependence was observed for all substances. The obtained results are shown in Figure 1.

At a concentration of 100 μM, no cytotoxic effect was observed on the test substances. Based on the dose–response curves, CC_50_ values (50% cytotoxic concentration) were calculated by nonlinear regression analysis (Table 2). According to the CC_50_ values, the most toxic substances were Si9 and Si10, with CC_50_ = 185 ± 4.4 μM and 173.3 ± 8.51 μM, respectively. The cytotoxicity of Si12 was significantly lower (*p* < 0.001), with CC_50_ = 879 ± 30.3 μM. The lowest cytotoxicity was observed in Si5 and Si4, with CC_50_ = 1272 ± 70.7 µM and 1219 ± 40.5 µM, respectively. CC_50_ values above 1000 μM are indicative of a low level of toxicity.

### 2.3. Antiproliferative Activity

**Figure 2:**
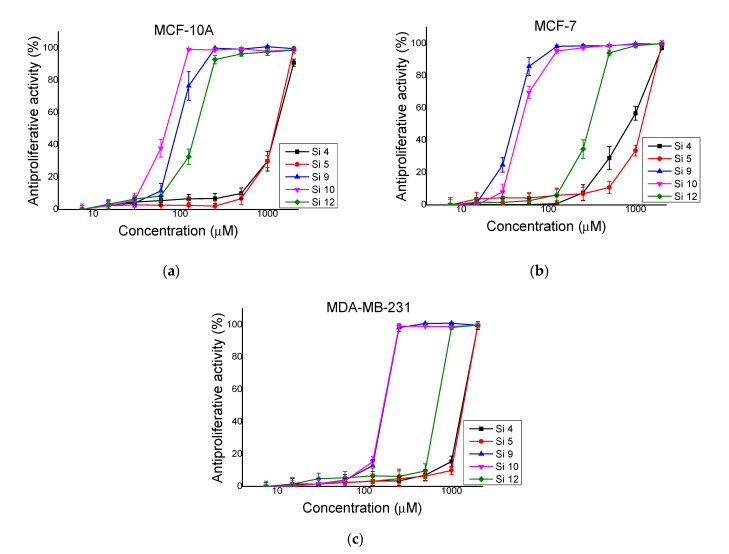
Antiproliferative activity of newly synthesized compounds. Dose–response curves assessment in (**a**) breast, (**b**) mammary gland type A adenocarcinoma (MCF-7) and (**c**) triple-negative breast cancer (MDA-MB-231). Values are means ± standard deviation from three independent experiments, *n* = 6.

The compounds were studied for antiproliferative activity by MTT dye-reduction assay. Cell cultures from different cell lines (MCF-10A, MCF-7 and MDA-MB-231) were incubated with the test substances at a concentration of 15 to 2000 μM for 72 h at 37 °C, 5% CO_2_ and 95% humidity. The antiproliferative activity expressed in % relative to the negative control was determined. The obtained results are shown in Figure 2. The IC_50_ (50% inhibitory concentration) values of the mean were calculated and presented in Table 2.

MCF-10A is a reliable model for normal human mammary epithelial cells, which serves as a control in experiments to determine antitumor activity.

In MCF-10A cells (Figure 2a), the least antiproliferative effect was caused by Si4 and Si5 (IC_50_ = 1326 ± 69.5 and IC_50_ = 1289 ± 38.1, respectively). In the cell line MCF-7, the strongest antitumor effect was shown by Si9 and Si10, with IC_50_ = 45 ± 4.2 and 50 ± 1.7, respectively (Figure 2b). The test substances were weakly active in the MDA-MB-231 cells (Figure 2c).

The IC_50_ values, found in MCF-10A, were used to calculate the selective index (SI), which assesses the potential of a substance to be used as an antitumor agent. We used the following formula to calculate the selective index: SI = IC_50_ of MCF-10A/IC_50_ of tumor cells. The highest selective index with respect to MCF-7 was shown by the substances: Si9 (SI = 2.19 ± 0.147), Si4 (SI = 1.51 ± 0.148) and Si10 (SI = 1.4 ± 0.072). With respect to MDA-MB-231, SI < 1 was observed for each of the test substances (Table 3).

### 2.4. Antimicrobial Activity

**Figure 3:**
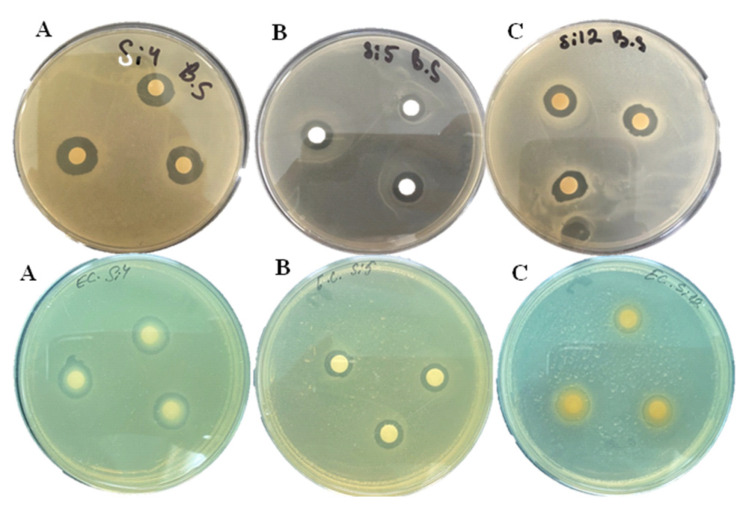
Antibacterial activity against *B. subtilis* 3562 (upper panel) and *E. coli* K12 407 (bottom panel) as test microorganisms for (**A**) Si4, (**B**) Si5 and (**C**) Si12. Antifungal activity of selected compounds is presented in Supplementary Materials.

The microbial cultures were placed in a thin layer on sterile plates. On sterile paper discs, 6 μL of 20 μM solutions in distilled water of the test compounds was placed. Further, the discs were incorporated into a thin layer of sterile plates (Figure 3). The studies were triplicates for each concentration, and the diameters of the obtained zones represent the average value of the three results. The obtained results are presented in Table 4.

### 2.5. Hydrolytic Stability Study

HPLC profiles of all target compounds at three different pH values that mimic human pH in the stomach (pH 2), blood plasma (pH 7.4) and small intestine (pH 9) are presented in the Supplementary Materials.

## 3. Discussion

Some of the main problems associated with currently used chemotherapeutics for the treatment of patients with various types of cancer are related to many undesired secondary effects of the body, as already mentioned in Section 1. In order to reduce damage to normal tissues, suboptimal doses of anticancer chemotherapeutics are often administered, which compromises the final effect. The specific structure of antimicrobial substances allows them to penetrate the cell membrane using different mechanisms [28,29,30]. Moreover, they are often conjugated to different molecules, including cytotoxic drugs, in order to deliver them to specific organs, tissues or cells [31,32,33]. As mentioned previously, many natural substances show anticancer potential due to the different targets that they attack. Some examples are curcumin produced by plants from the *Curcuma longa* species [34,35], caffeine contained in the beans of *Coffea arabica* and *Coffea canephora* [36], caffeic acid (Caf) [37,38,39,40,41], etc. In addition, 1,8-naphthalimide (Npht) and its differently substituted derivatives [27,42,43,44,45] are synthetic compounds with proven anticancer properties, some of them already used in medical practice [46,47]. In 2012, Ma et al. used data available in the literature to determine the activity and penetration ability of (KLAKLAK)_2_ and its D-amino acid containing analog D(KLAKLAK)_2_ in order to produce conjugates with higher potential to penetrate the cell membrane and bring a second pharmacophore to tumor cells [48]. As a result of their study, they found that a conjugated bioactive peptide with D(KLAKLAK)_2_ is an efficient antitumor agent both in vitro and in vivo. Taking into account the following datum:

- Our previous positive results obtained by introducing an β-Ala moiety to the shortened analogs of (KLAKLAK)_2_-NH_2_ consisting of high antiproliferative activity and selectivity to MCF-7 cells of the structures KLβAKLβAK-NH_2_ and Caf-KLβAKLβAK-NH_2_ [25];
- The positive effects of introducing β-amino acid for further biological activity, described in the review of Cabrele et al.;
- The results reported by Ma and coworkers

We synthesized two groups of compounds. The first group consists of analogs of the antimicrobial peptide (KLAKLAK)_2_-NH_2_ containing Ala or β-Ala with the general formula (LysLeuXLysLeuXLys)_2_-NH_2_, where X is Ala or β-Ala. The second group comprises bioconjugates of (KLAKLAK)_2_-NH_2_ or modified with unnatural amino acids β-Ala (KLAKLAK)_2_-NH_2_ with NphtG/Caf. The new structures have general formula (YLysLeuXLysLeuXLys)_2_-NH_2_, where X is Ala or β-Ala, and Y is NphtG or Caf.

Biological activity (cytotoxicity and antiproliferative activity) was determined on all these newly synthesized molecules. The CC_50_ value is indicative of the cytotoxic potential of a substance. For parent compound Si1 (KLAKLAK)_2_-NH_2_ in murine embryonic fibroblasts (BALB 3T3), CC_50_ = 365.3 ± 4.08 [25]. When Ala was replaced with β-Ala in the structure Si5, a significant (*p* < 0.001) decrease in cytotoxicity (CC_50_ = 1272.0 ± 70.70) was observed. At the same time, a decrease in antiproliferative activity was also observed. The addition of the second pharmacophore 1,8-NphtG- to (KLβ-AKLβ-AK)_2_-NH_2_ to compound Si4 did not lead to a statistically significant (*p* > 0.05) change in biological activity. In contrast, the introduction of a Caf- group to Si12 significantly (*p* < 0.001) increased cytotoxicity and antiproliferative activity. The strongest antiproliferative effect against the MCF-7 cell line was observed when the 1,8-NphtG- and Caf- groups were added to the peptide containing only natural amino acids (Si9 and Si10 with IC_50_ values of 45.2 ± 4.15 and 50.5 ± 1.66, respectively). In addition, an increased selectivity index was observed for Si9 and Si10 (SI = 2.19 and 1.44, respectively).

The analysis of the results presented in this article and their comparison with the results of our previous studies show that the length of the peptide chain is of key importance for antiproliferative activity [25]. There is probably an optimal length of the peptide at which the highest level of antitumor activity is observed. This could explain the increase in the antitumor activity of the short peptide composed of seven amino acids (KLAKLAK-NH_2_) when amino acid Ala is replaced by unnatural analog β-Ala (KLβ-AKLβ-AK-NH_2_), i.e., the length of the peptide is increased with several methylene groups. Conversely, when amino acid Ala is replaced with β-Ala, in the longer peptide composed of 14 amino acids (KLβ-AKLβ-AK)_2_-NH_2_), the optimal length is exceeded, and as a result, a reduction in antiproliferative activity is observed.

The current study on antibacterial properties revealed that samples Si4, Si5 and Si12 containing β-Ala in their structures exhibit good antimicrobial activity. An inhibition zone of 16 mm is observed for the compound S4 against *E. coli* K12 407, and against *B. subtilis* 3562, the inhibition zone is 15.83 mm. The samples containing compound Si10, with α-Ala in their structure, also showed very good antimicrobial activity comparable to both test strain microorganisms with an inhibition zone against *E. coli* K12 407 of 13.33 mm and against *B. subtilis* 3562 of 12.66 mm. The highest inhibition zone of 20.66 mm against *C. albicans* 74 was formed around sample Si9. Samples Si4 and Si10 also showed good antimicrobial activity, while samples Si5 and Si12 did not show any antifungal activity. Our previous study on shortened analogs of KLAKLAK-NH_2_, also containing Ala and β-Ala, revealed that the samples with α-Ala had weak antibacterial activity only against *E. coli* K12 407, while against *B. subtilis 3562* and *C. albicans* 74, some antimicrobial activity was observed [25].

The hydrolytic stability of all newly synthesized molecules was monitored for a period of 72 h in model buffer solutions at three different pH values that mimic the pH in the stomach (pH 2), blood plasma (pH 7.4) and small intestine (pH 9) of the human body. The designed changes in the structures, i.e., C-terminal amide modification, as well as the introduction of a β-Ala moiety, lead to complete stability of the target compounds during the test period in all model conditions.

## 4. Conclusions

Taking into account our previous data published in [25] and the results in the current study, we can conclude that the length of the molecule chain plays a crucial role in antiproliferative and antimicrobial activity. Compounds Si9 1,8-NphtG-(KLAKLAK)_2_-NH_2_ and Si10 Caf-(KLAKLAK)_2_-NH_2_, combining the parent molecule with the second pharmacophores 1,8-NphtG- and Caf-, show the best activity against MCF-7 tumor cell lines, together with good SI. In addition, they have very good antifungal activity against the used model strain *C. albicans* 74. These properties are accompanied by complete hydrolytic stability during a 72 h period.

The compounds Si4 1,8-NphtG-(KLβ-AKLβ-AK)_2_-NH_2_ and Si5 (KLβ-AKLβ-AK)_2_-NH_2_ containing unnatural amino acid β-Ala in their structure combine low cytotoxicity and high antibacterial activity, accompanied by complete hydrolytic stability during a 72 h period. The compound Caf-(KLβ-AKLβ-AK)_2_-NH_2_ (Si12), also containing β-Ala in its structure but the Caf-group as a second pharmacophore, exhibits good antimicrobial activity against *E. coli* K12 407 and *B. subtilis* 3562 in combination with very good antiproliferative and cytotoxic properties as well as hydrolytic stability.

All mentioned compounds could be useful in medical practice as anticancer or antimicrobial agents.

## 5. Materials and Methods

### 5.1. Synthesis and Analytical Data

All specifically protected amino acids ^α^*N*-Fmoc-Lys(Boc)-OH, Fmoc-β-Ala-OH, Fmoc-Leu-OH, Fmoc-Rink amide MBHA resin, activation agents *N*,*N*,*N*′,*N*′-tetramethyl-*O*-(1*H*-benzotriazol-1-yl)uronium hexafluorophosphate (HBTU), *N*,*N*′-diisopropylcarbodiimide (DIC) or benzotriazol-1-yloxy)tripyrrolidinophosphonium hexafluorophosphate (PyBOP), trifluoroacetic acid (TFA) and scavenger triisopropylsilane (TIS) were purchased from Iris Biotech (Wunsiedel, Germany). The used solvents DMF and DCM, as well as caffeic acid (Alfa Aesar, Ward Hill, MA, USA), are from Valerus (Sofia, Bulgaria). 1,8-Naphthalanhydride is from Sigma-Aldrich (Ansbach, Germany). All reagents and solvents were used without any additional treatment.

A conventional Fmoc(9-fluorenylmethoxycarbonyl)/Ot-Bu solid-phase peptide synthesis strategy on Rink amide MBHA resin was applied for the synthesis of the target compounds. HBTU, DIC or PyBOP were used for amino acid activation. The coupling reactions were performed using amino acid/HBTU (or PyBOP)/HOBt/DIEA/resin at a molar ratio of 3/3/3/9/1 or amino acid/DIC/resin at a molar ratio of 3/3/1. The Fmoc group was deprotected by treatment with 20% piperidine/DMF solution. The coupling and deprotection reactions were monitored by means of the Kaiser test. The cleavage of the final peptides from the resin was performed using a mixture of 95% trifluoroacetic acid (TFA), 2.5% triisopropylsilane (TIS) and 2.5% distilled water. All peptides were obtained as a filtrate in TFA and precipitated with cold dry diethyl ether. The precipitate was filtered and subjected to analysis.

Peptide purity was monitored on a 100 mm × 4.6 mm RP-HPLC Agilent Poroshell 120 column using the Shimadzu LC-MS/MS 8045 system at a mobile phase flow rate of 0.30 mL/min, a column temperature of 40 °C and a linear binary two-phase gradient, Mobile Phase A: H_2_O (10% AcCN; 0.1% HCOOH) and Mobile Phase B: AcCN (5% H_2_O; 0.1% HCOOH), with the gradients of both phases over time presented in Table 5.

The compounds structures were checked by electrospray ionization mass spectrometry in SCAN regime/ESI+ ionization mode with the parameters presented in Table 6.

The optical rotation was measured on a Polamat A automatic standard polarimeter, Carl Zeiss, Jena, at c = 1 in water. Melting points were monitored on a standard Kofler hot-stage microscope. All analytical data are summarized in Table 1.

### 5.2. Cell Cultures

Two human mammary carcinoma cell lines MCF-7 (ER+, PR+ and Her-2−) and MDA-MB-231 (ER−, PR− and Her-2−) were used as models for breast cancer. The cell lines BALB 3T3 (mouse embryonic fibroblasts) and MCF-10A (human breast epithelial cell line) were used as models for healthy tissue. Cell lines were obtained from the American Type Culture Collection (ATCC, Manassas, Virginia, USA). Cells were cultured in Dulbecco Modified Eagle’s medium (DMEM) supplemented with 10% (*v*/*v*) fetal bovine serum, 100 U/mL penicillin and 0.1 mg/mL streptomycin (Sigma-Aldrich, Schnelldorf, Germany) in an incubator at 37 °C, 5% CO_2_ and 95% humidity. Plastic flasks of 25 cm^2^ (Biologix, Lenexa, KS, USA) were used to grow the cells.

### 5.3. In Vitro Cytotoxicity Testing (3T3 NRU Test)

The cytotoxicity testing was performed as described by Borenfreund et al. [49] and the latest modification of the validated BALB/3T3 clone A31 Neutral Red Uptake Assay (3T3 NRU test) [50]. BALB/3T3 clone A31 mouse embryo cells were grown as a monolayer in 75 cm^2^ tissue culture flasks in high-glucose DMEM (4.5 g/L), supplemented with 10% FBS and antibiotics. Cultures were maintained at 37.5 °C in a humidified atmosphere under 5% CO_2_. Cells were plated at a density of 1 × 10^4^ cells in 100 μL culture medium in each well of 96-well flat-bottomed microplates and allowed to adhere for 24 h before treatment with test compounds, dissolved in DMSO and further diluted in culture medium. A wide concentration range was applied (from 15 to 2000 μM), and the cells were incubated for an additional 24 h. After treatment with Neutral Red medium, washing and application of the ethanol/acetic acid solution (NR Desorb), the absorption was measured on a TECAN microplate reader at wavelength 540 nm. The data obtained are on average from three independent experiments ± SD, *n* = 6.

### 5.4. In Vitro Antiproliferative Activity

The antiproliferative activity testing was performed on cell cultures from several human cell lines using the standard MTT dye-reduction assay, described by Mosmann [51]. The assay is based on the metabolism of the tetrazolium salt 3-(4,5-dimethylthiazol-2-yl)-2,5-diphenyltetrazolium bromide (MTT) to insoluble formazan by mitochondrial reductases. The formazan concentration can be determined spectrophotometrically. The measured absorption is an indicator of cell viability and metabolic activity. The following cell lines were used in the experiments: mammary gland type A adenocarcinoma ER+, PR+ and HER2− (MCF-7); triple-negative breast cancer ER−, PR−, and HER2− (MDA-MB-231); and breast, nontumorigenic epithelial cell line (MCF-10A). The cell lines were routinely grown as monolayers in 75 cm^2^ tissue culture flasks under standard conditions (described above). Cells were plated at a density of 1 × 10^3^ cells in 100 µL in each well of 96-well flat-bottomed microplates and allowed to adhere for 24 h before treatment with test compounds. A concentration range from 15 to 2000 μM was applied for 72 h. Formazan absorption was registered using a microplate reader at λ = 540 nm. Antiproliferative activities were expressed as IC_50_ values (concentrations required for 50% inhibition of cell growth), calculated using nonlinear regression analysis (GraphPad Prism 4 Software, https://www.graphpad.com).

The statistical analysis included the application of one-way ANOVA, followed by Bonferroni’s post hoc test. The test is used to compare the results (IC_50_ values) obtained for all tested peptides and determine statistically significant differences. *p* < 0.05 was accepted as the lowest level of statistical significance. All results are presented as mean ± SD.

### 5.5. Antimicrobial Assay

All newly synthesized derivatives of (KLAKLAK)_2_-NH_2_ at concentrations of 20 μM in distilled water were tested against model strains *E. coli* NBIMCC K12 407, *B. subtilis* NBIMCC 3562 and *C. albicans* NBIMCC 74. The strains were obtained from the National Bank for Industrial Microorganisms and Cell Cultures (NBIMCC, Sofia, Bulgaria). Exponential cultures (1 × 10^7^ cfu/mL) of strains *E. coli* K12 407 were obtained in Luria-Bertani (LB, HiMedia, Mumbai, India); for *B. subtilis* 3562, an initial bacterial concentration of 1 × 10^6^ cfu/mL was obtained in nutrient broth (NB, HiMedia, Mumbai, India); and for *C. albicans* 74, and initial bacterial concentration of 1 × 10^5^ cfu/mL was obtained in yeast mold (YM) medium. Cultivation was performed in an ES-20/60 incubator shaker (Biosan, Riga, Latvia) at 30 °C for *B. subtilis* 3562 and at 37 °C for *E. coli* K12 407 and *C. albicans* 74 with shaking at 220 rpm for 24 h. The agar diffusion method was applied to determine the antimicrobial activity of the tested compounds. LB/NB/YM plates were inoculated by spreading 100 μL of each microbial culture on their surface, followed by incubation at 30/37 °C for penetration of suspension into the agar. After 30 min, sterile paper discs (6 mm in diameter) were soaked with the tested compounds in amounts of 6 µL and placed on the surface of agar Petri dishes. The plates were incubated at 30/37 °C for 24 h. Antimicrobial activity was assessed by measuring the diameter of the obtained inhibition zones. Sterile paper discs soaked with water were used as blank controls. Mean values were calculated by performing the experiments in triplicates.

### 5.6. Hydrolytic Stability

Three different pH values that mimic human pH in the stomach, blood plasma and small intestine were selected for investigation of hydrolytic stability of newly synthesized compounds. Model solutions used for determination of hydrolytic stability were prepared according to the European Pharmacopoeia, 6th Edition, as follows:

(i) Buffer with pH 2.0–6.57 g KCl was dissolved in water (CO_2_ free), and 119.0 mL 0.1 mol/L HCl was added. The obtained solution was made up to 1000.0 mL with dH_2_O;
(ii) Buffer with pH 7.4–2.38 g Na_2_HPO_4_, 0.19 g KH_2_PO_4_ and 8.0 g NaCl was dissolved in dH_2_O. The obtained solution was made up to 1000.0 mL with dH_2_O.
(iii) Buffer with pH 9.0–1000.0 mL of solution I was mixed with 420.0 mL of solution II. Solution I: 6.18 g of H_3_BO_3_ was dissolved in 0.1 mol/L KCl, and it was made up to 1000.0 mL with the same solvent; Solution II: 0.1 mol/L NaOH.

HPLC MODEL 550 A of KONIK-TECH, Sant Cugat del Vallès, Spain, with photodiode array detectors and a Hypersil BDS C8, 150 × 4.6 mm, 5 μm column was used for monitoring the hydrolytic stability: mobile phase: 50:50 acetonitrile–water; elution rate: 0.9 mL/min; room temperature; scanning wavelength: 255 nm for all compounds, except for Si5, where it was 190 nm; injected volume: 20 µL.

## Figures and Tables

**Table 1:** Structure and analytical data for newly synthesized compounds.

Code	Structure	Molecular Formula	MM _exact_	[M + H]^+^ Observed	[M + Na]^+^ Observed	t_R_ (min)	M.p. (°C)	$\alpha_{546}^{20}$ (°) *	Chromatographic Purity (%)
Si4	1,8-NphtG-(KLβ-AKLβ-AK)_2_-NH_2_	C_85_H_142_N_22_O_18_	1759.08	1760.17	-	2.497	100–102	−56	99
Si5	(KLβ-AKLβ-AK)_2_-NH_2_	C_71_H_135_N_21_O_15_	1522.05	1523.85	-	2.449	120–122	−108	100
Si9	1,8-NphtG-(KLAKLAK)_2_-NH_2_	C_85_H_142_N_22_O_18_	1759.08	1760.65	1783.45	3.350	136–138	−110	96
Si10	Caf-(KLAKLAK)_2_-NH_2_	C_81_H_145_N_21_O_17_	1685.15	1686.45	1686.45	1.357	122–124	−132	98
Si12	Caf-(KLβ-AKLβ-AK)_2_-NH_2_	C_81_H_145_N_21_O_17_	1685.15	1687.35	1709.35	1.203	160–162	−82	100

* Water (c = 1).

**Table 2:** Cytotoxic and antiproliferative activity of the studied substances expressed by IC_50_ values of the mean ± SD.

Code	IC_50_ of Mean ± SD (μM)			
	Cytotoxicity	Antiproliferative Activity		
	BALB 3T3	MCF-10A	MCF-7	MDA-MB-231
Si 1 *	315.3 ± 4.076	154.0 ± 6.53	124.10 ± 8.12	746.5 ± 7.6
Si 4	1219.0 ± 40.51	1326.0 ± 69.46	881.0 ± 80.46	1411.0 ± 26.25
Si 5	1272.0 ± 70.70	1289.0 ± 38.13	1254.0 ± 34.07	1448.0 ± 15.73
Si 6 **	>4000	>2000	>2000	>2000
Si 9	185.4 ± 4.40	99.1 ± 5.59	**45.2 ± 4.15**	179.3 ± 2.92
Si 10	173.3 ± 8.51	72.5 ± 4.69	**50.5 ± 1.66**	176.6 ± 2.79
Si 12	879.8 ± 30.27	160.8 ± 7.39	313.5 ± 18.78	727.7 ± 15.30

* Si1 ((KLAKLAK)_2_-NH_2_); ** Si6 (KLAKLAK-NH_2_). Data are already published in [25].

**Table 3:** Selective index calculated by IC_50_ values of MCF-10A cell line.

Code	SI ± SD *	
	MCF-7	MDA-MB-231
Si 1	1.243 ± 0.052	0.206 ± 0.007
Si 4	**1.505 ± 0.148**	0.940 ± 0.049
Si 5	1.028 ± 0.033	0.890 ± 0.033
Si 6	-	-
Si 9	**2.192 ± 0.147**	0.553 ± 0.037
Si 10	**1.436 ± 0.072**	0.411 ± 0.023
Si 12	0.513 ± 0.019	0.221 ± 0.012

* Values are means ± standard deviation from three independent experiments, n = 6

**Table 4:** Antimicrobial activity expressed as inhibition zones size (mm) against several test microorganisms.

Code	Structures	* Escherichia coli K12 407	* Bacillus subtilis 3562	* Candida albicans 74
Si 4	1,8-NphtG-(KLβ-AKLβ-AK)_2_-NH_2_	16.00 ± 0.43	15.83 ± 0.38	14.33 ± 0.36
Si 5	(KLβ-AKLβ-AK)_2_-NH_2_	11.83 ± 0.27	11.16 ± 0.25	0
Si 9	1,8-NphtG-(KLAKLAK)_2_-NH_2_	11.33 ± 0.26	13.50 ± 0.34	20.66 ± 0.45
Si 10	Caf-(KLAKLAK)_2_-NH_2_	13.33 ± 0.33	12.66 ± 0.31	17.66 ± 0.41
Si 12	Caf-(KLβ-AKLβ-AK)_2_-NH_2_	12.33 ± 0.30	12.50 ± 0.31	0

* Data are expressed as means of 3 replicates ± SD.

**Table 5:** Gradient of mobile phases over time.

Time (Min)	Mobile Phase A (%)	Mobile Phase B (%)
0.01	80	20
10.00	5	95
15.00	5	95
15.50	80	20
22.00	80	20

**Table 6:** MS parameters.

Parameter	Value
Nebulizing gas flow	3 L/min
Heating gas flow	10 L/min
Interface temperature	350 °C
DL temperature	200 °C
Heat block temperature	400 °C
Drying gas flow	10 L/min

## Data Availability

The data presented in this study are available from the authors.

## Supplementary Materials

Supplementary materials are available online, contain HPLC/MS profiles of target compounds, HPLC profiles of hydrolytic stability determination and plates of selected compounds with *Candida albicans* studies.

## Sample Availability

Small quantities of all target compounds are available from authors at the University of Chemical Technology and Metallurgy.
