# Supplementary material for: Synthesis and Biological Studies on (KLAKLAK)2-NH2 Analog Containing Unnatural Amino Acid β-Ala and Conjugates with Second Pharmacophore
Source: Molecules. 2021 Dec 2;26(23):7321. doi: 10.3390/molecules26237321 (PMC8658989; doi:10.3390/molecules26237321)
Supplement: Supplementary file 1 [file molecules-26-07321-s001.zip › molecules-1471357-supplementary.pdf]

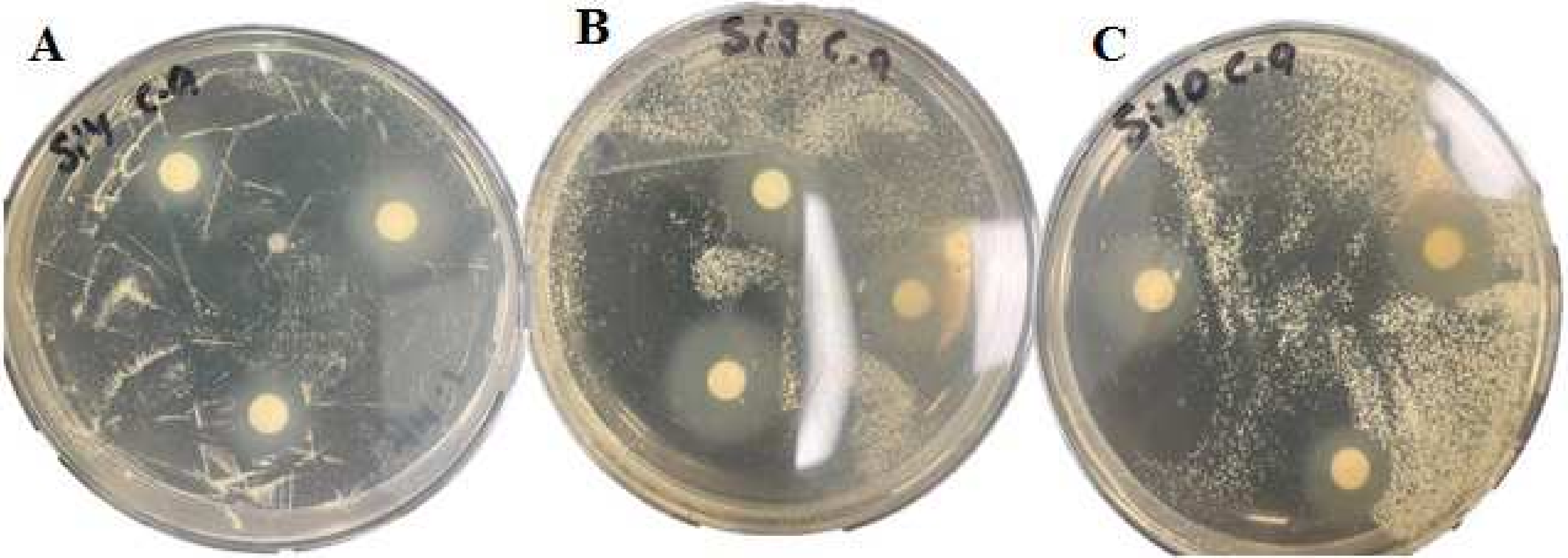

Antimicrobial activity expressed as inhibition zones size (mm) against *Candida albicans* 74 for compounds Si4 (A), Si9 (B), Si10 (C).

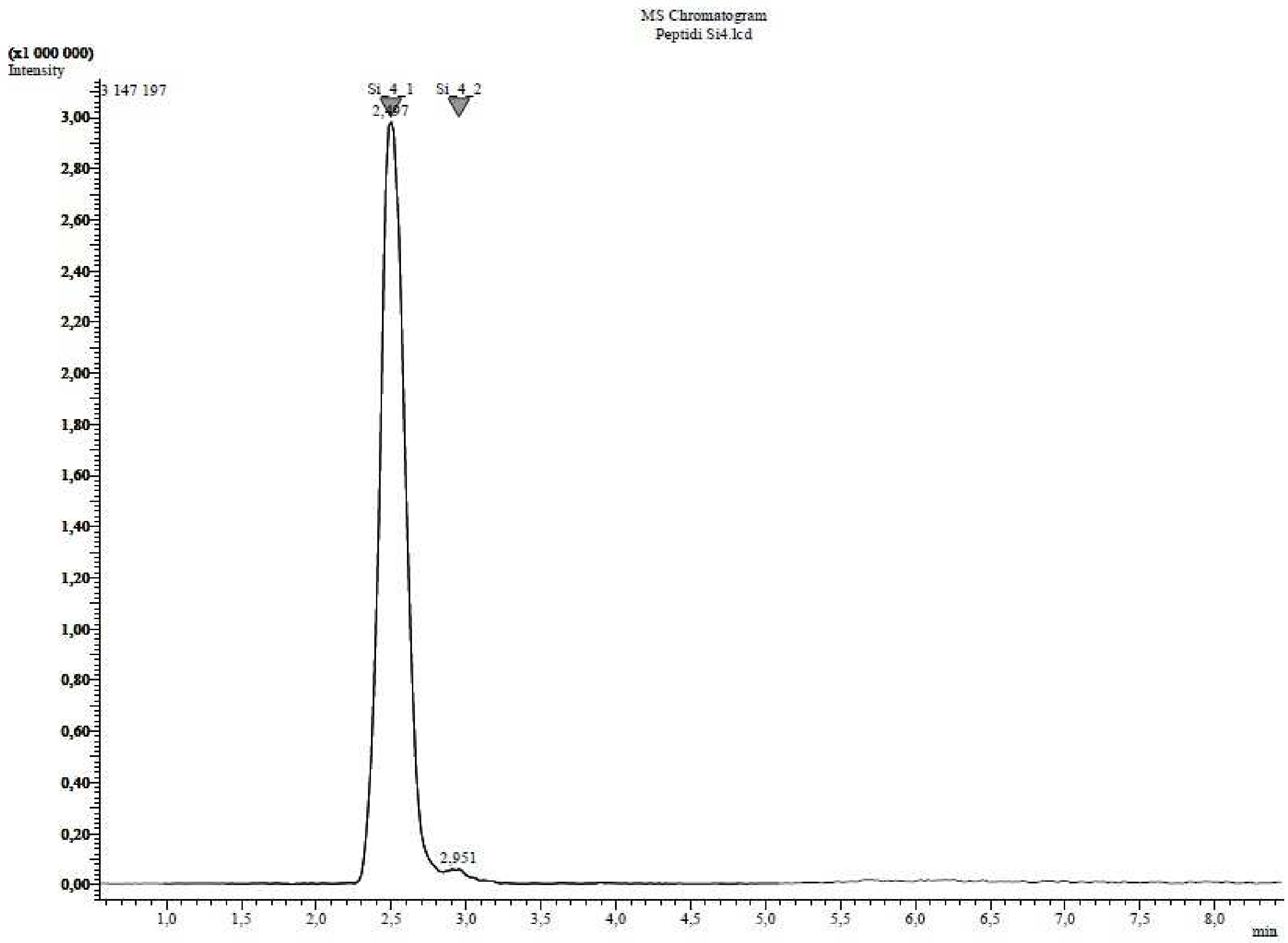

C:\LabSolutions\Data\Data\Peptidi\19062019\Si4.lcd

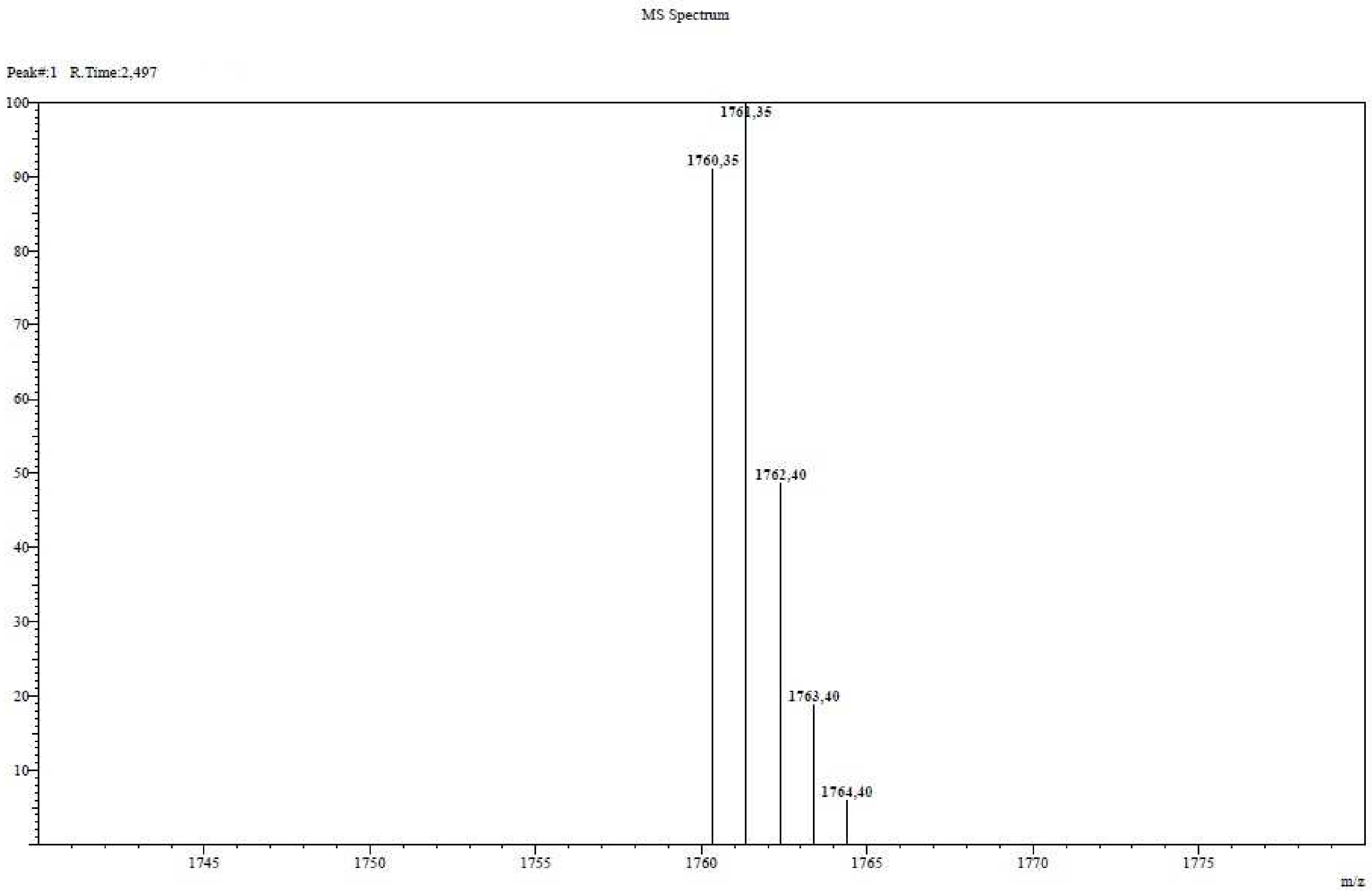

C:\LabSolutions\Data\Data\Peptidi\19062019\Si4.lcd

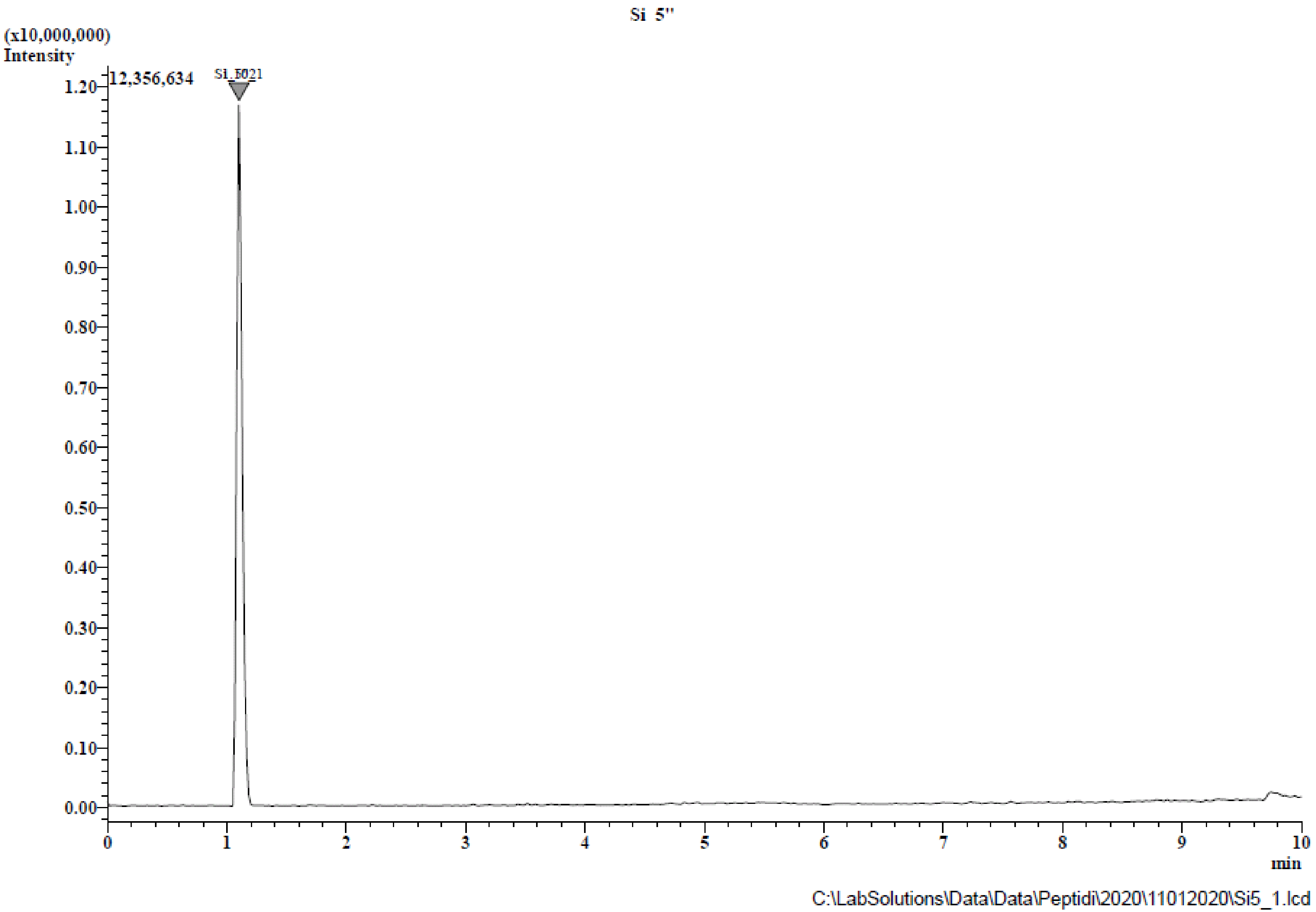
Si 5''
(x10,000,000)
Intensity
12,356,634
S1_B021
1.20
1.10
1.00
0.90
0.80
0.70
0.60
0.50
0.40
0.30
0.20
0.10
0.00
0
1
2
3
4
5
6
7
8
9
10
min
C:\LabSolutions\Data\Data\Peptidi\2020\11012020\Si5_1.lcd

MS Spectrum

Peak#:1 R.Time:1.102

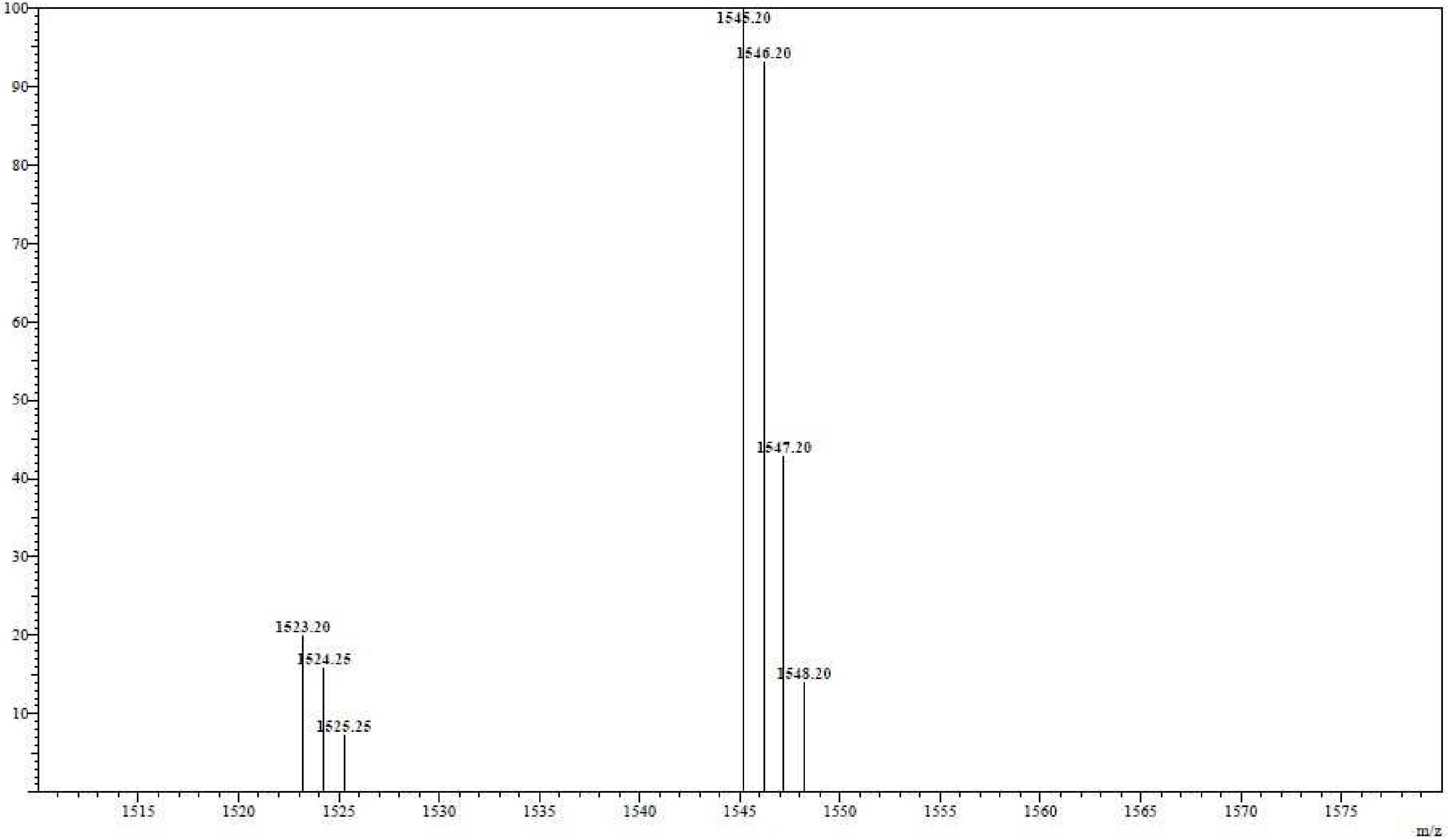

C:\LabSolutions\Data\Data\Peptidi\2020\11012020\Si5_1.lcd

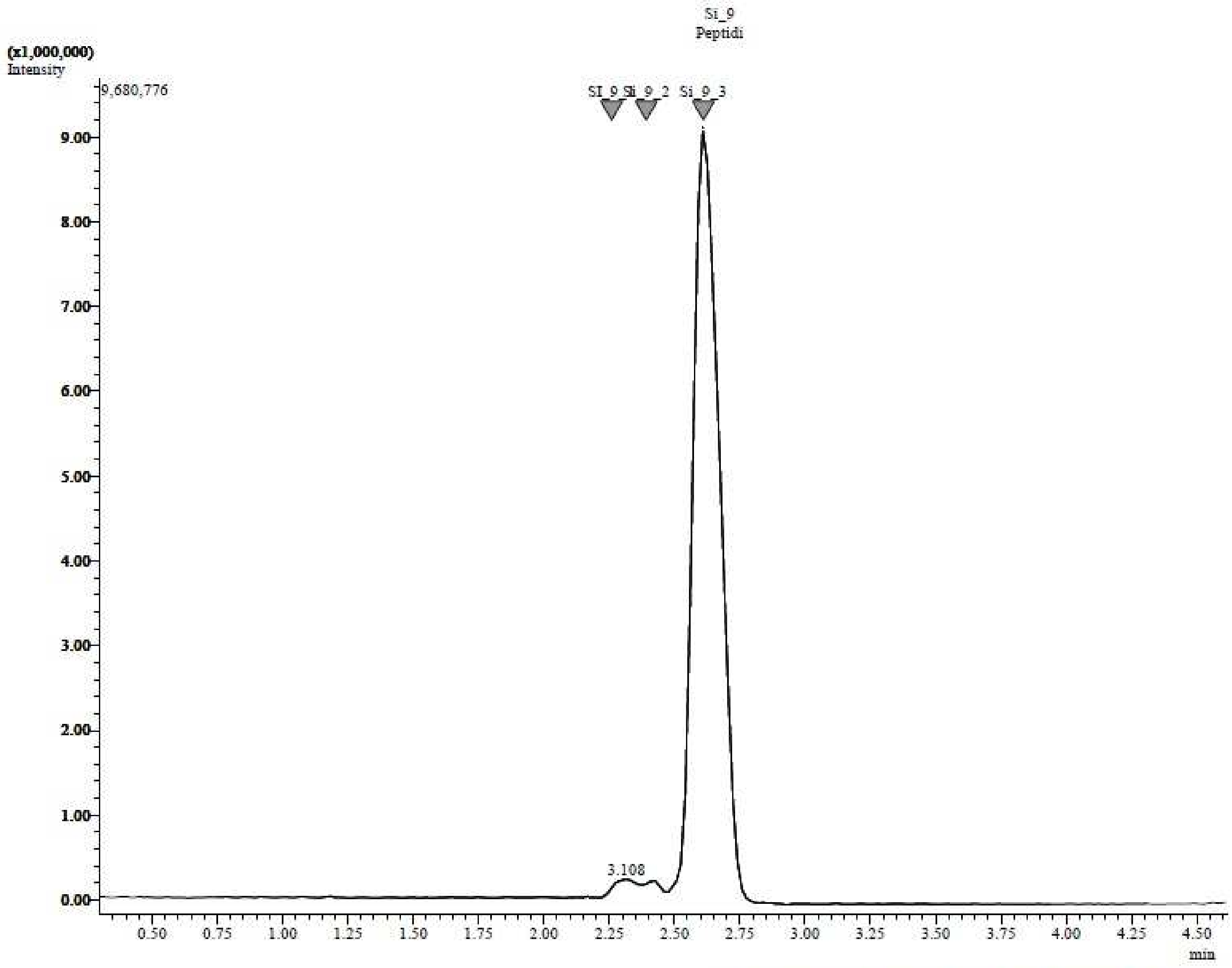

C:\LabSolutions\Data\Data\Peptidi\25102019\Si_9_1.lcd

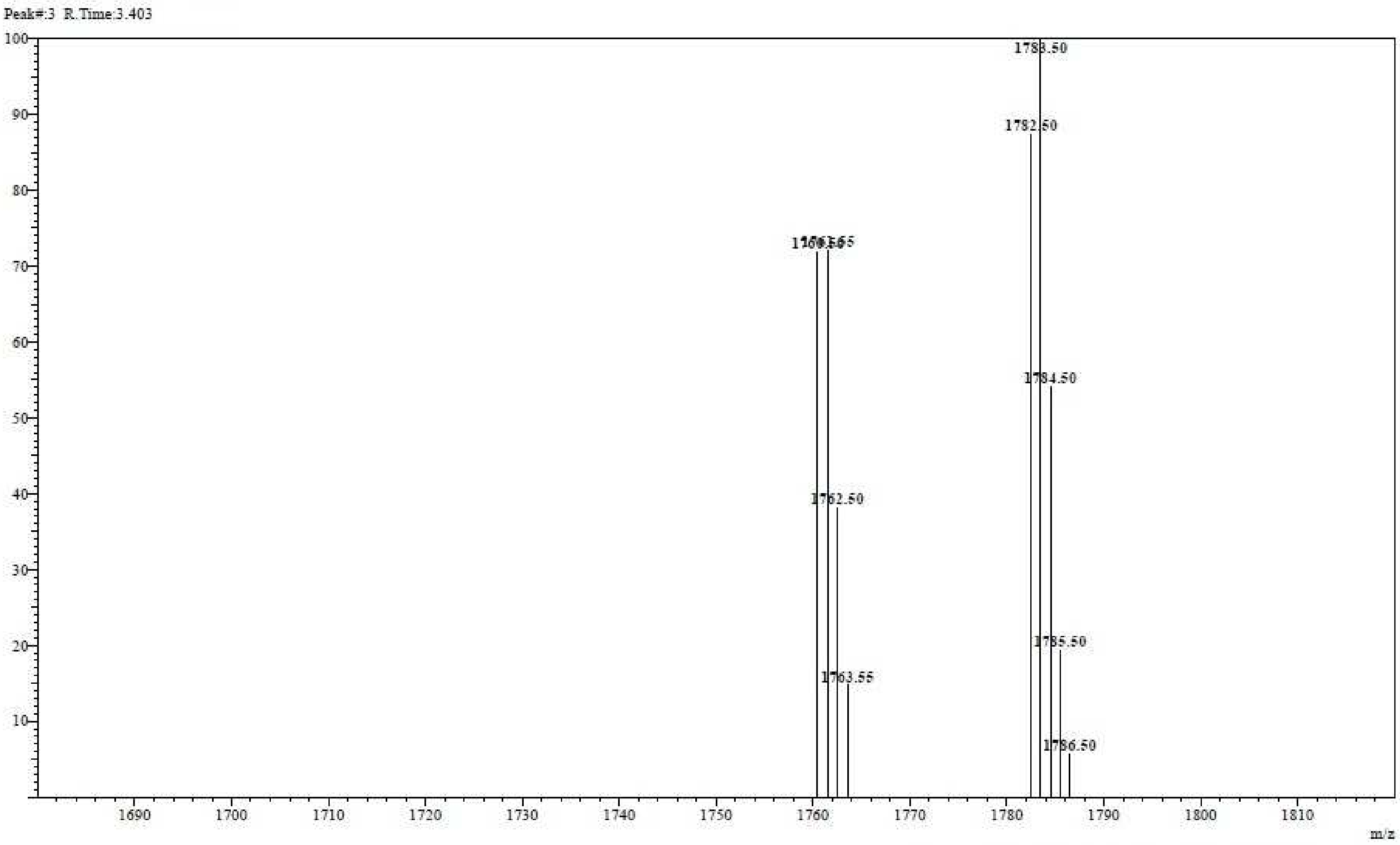

C:\LabSolutions\Data\Data\Peptidi\25102019\Si_9_1.lcd

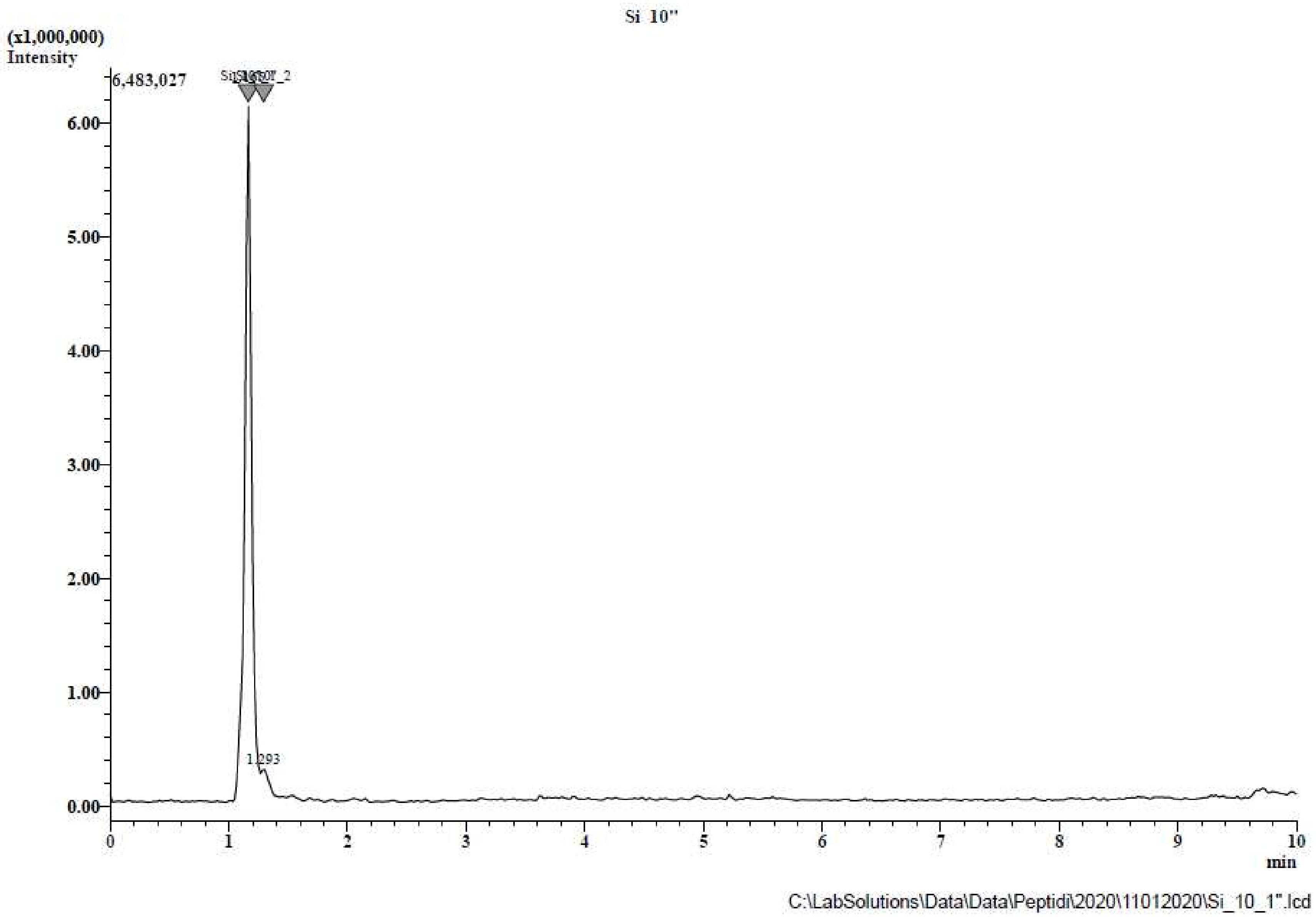

Si 10"
(x1,000,000)
Intensity
6,483,027
6.00
5.00
4.00
3.00
2.00
1.00
0.00
1.293
0
1
2
3
4
5
6
7
8
9
10
min
C:\LabSolutions\Data\Data\Peptidi\2020\11012020\Si_10_1".lcd

MS Spectrum

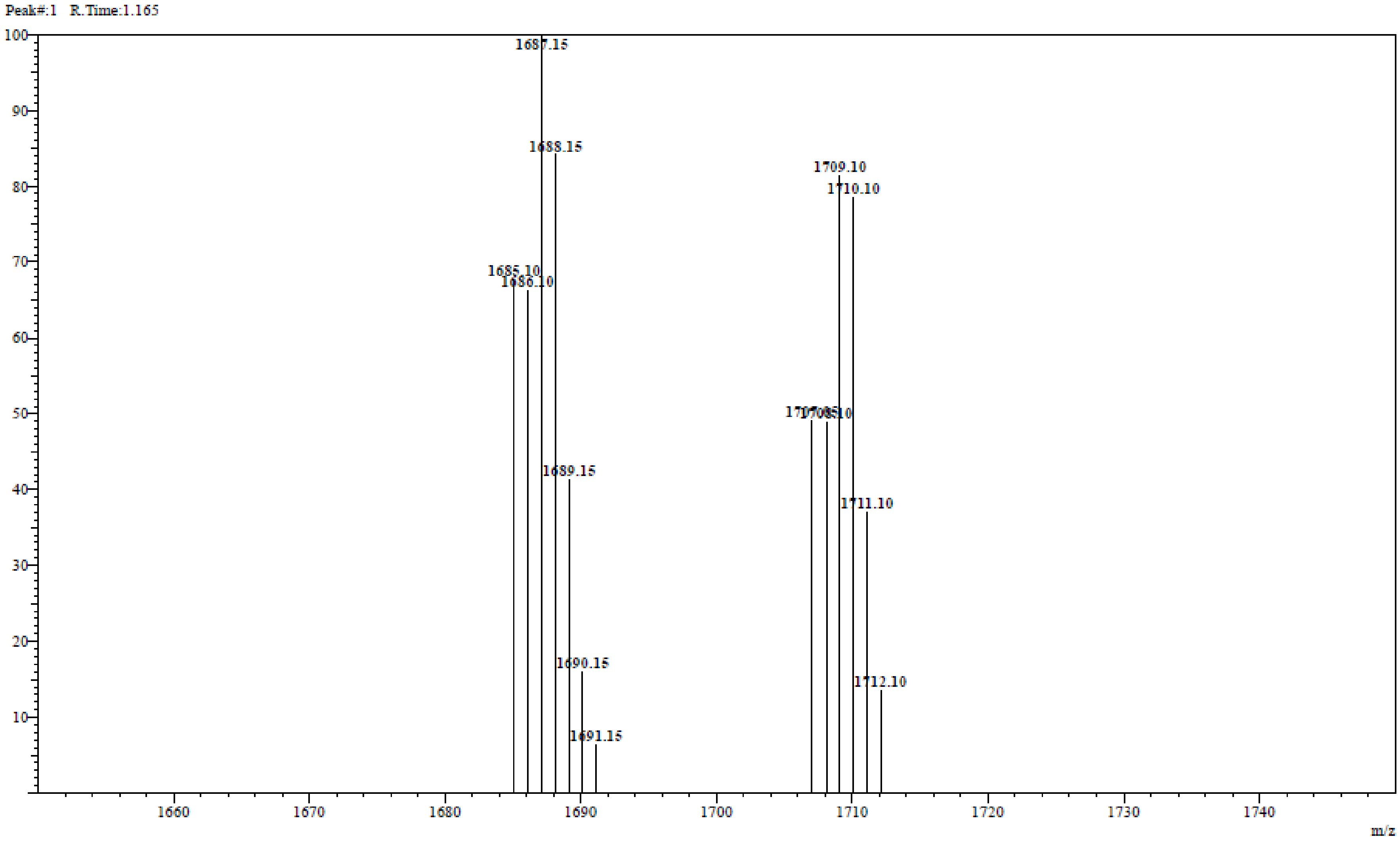

C:\LabSolutions\Data\Data\Peptidi\2020\11012020\Si_10_1".lcd

Si 12

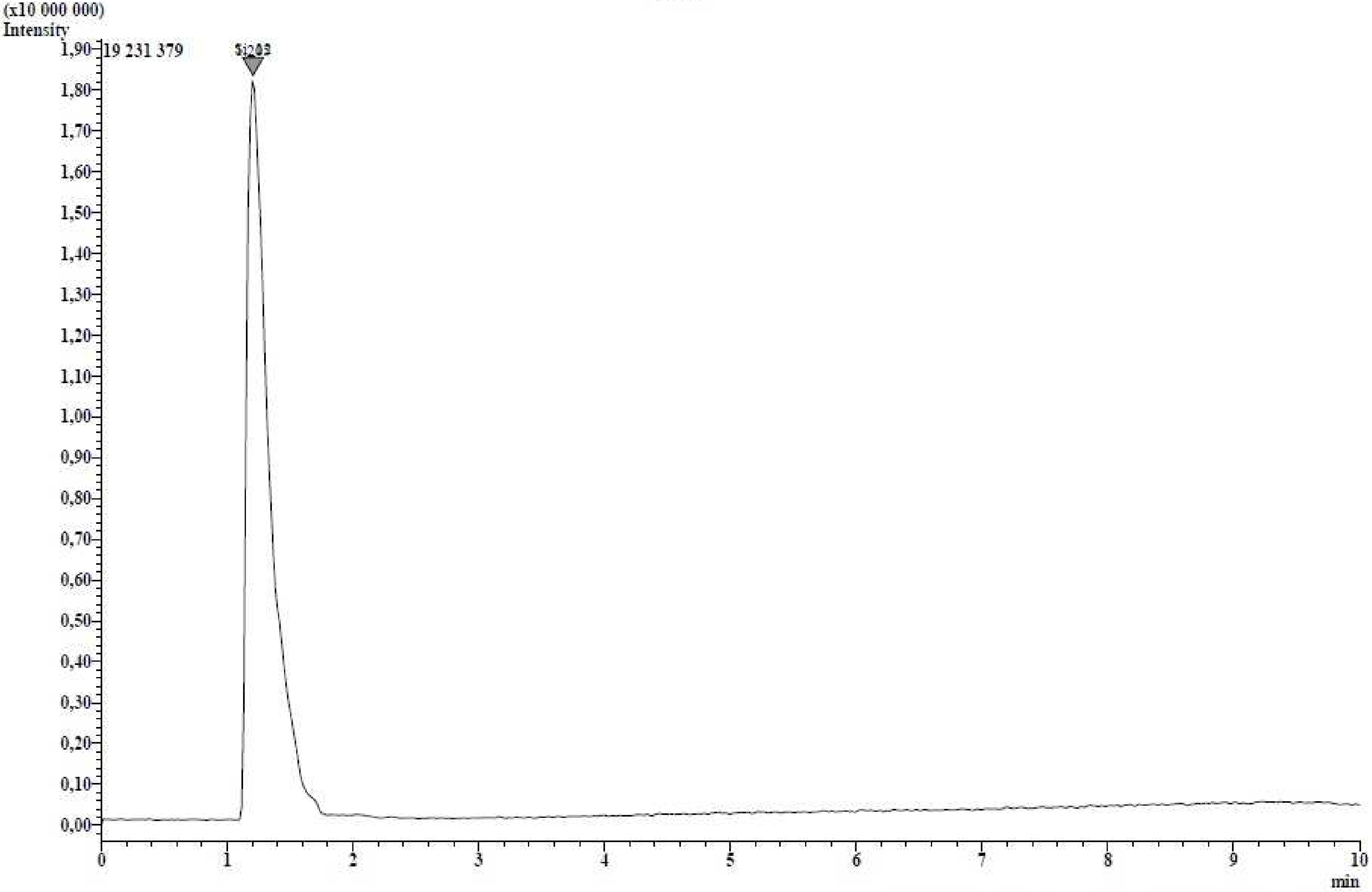

C:\LabSolutions\Data\Data\Peptidi\2392019\Si_12_1.lcd

MS Spectrum

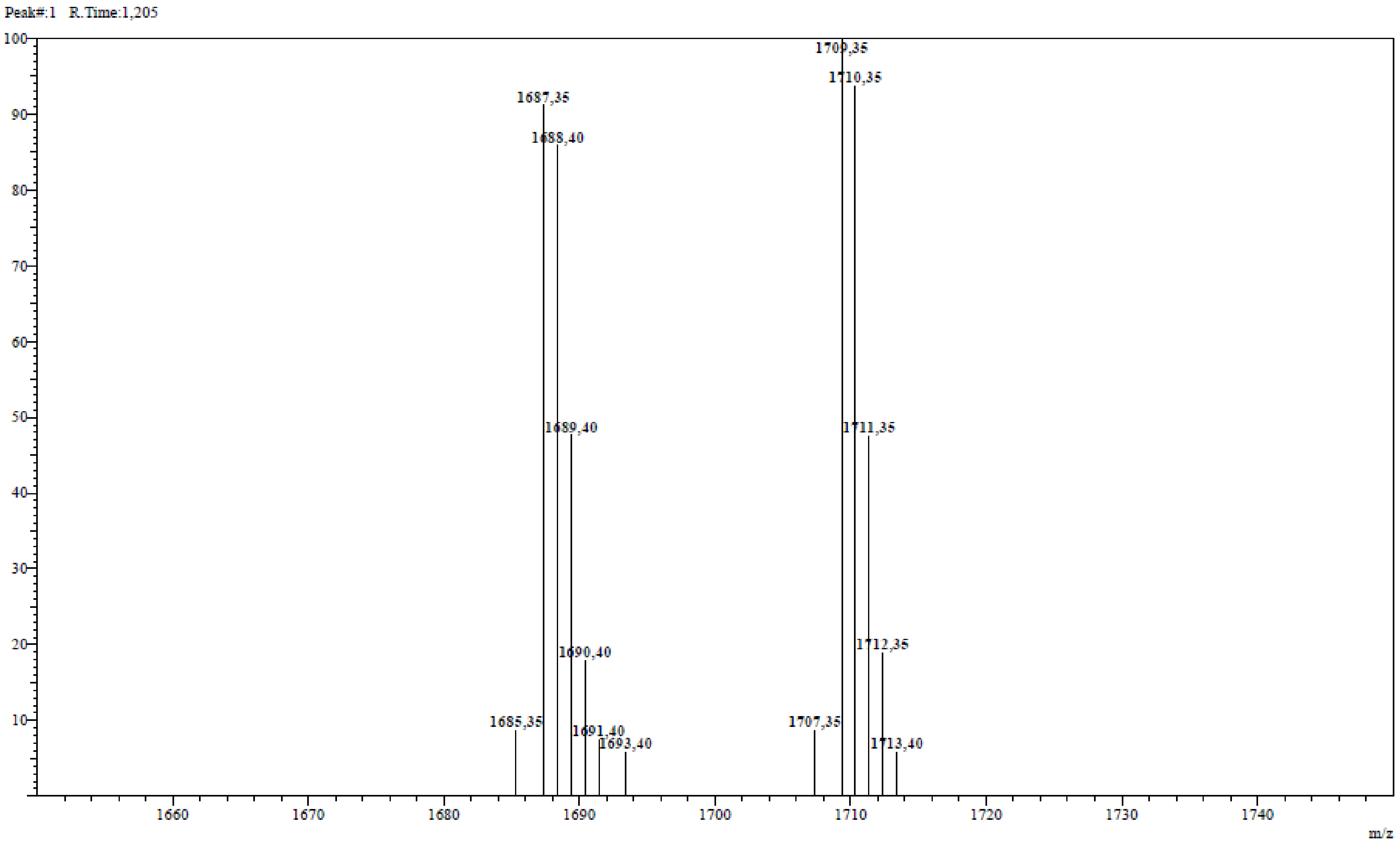

C:\LabSolutions\Data\Data\Peptidi\2392019\Si_12_1.lcd

HPLC profiles of aimed compounds at different pH

pH2

Si4

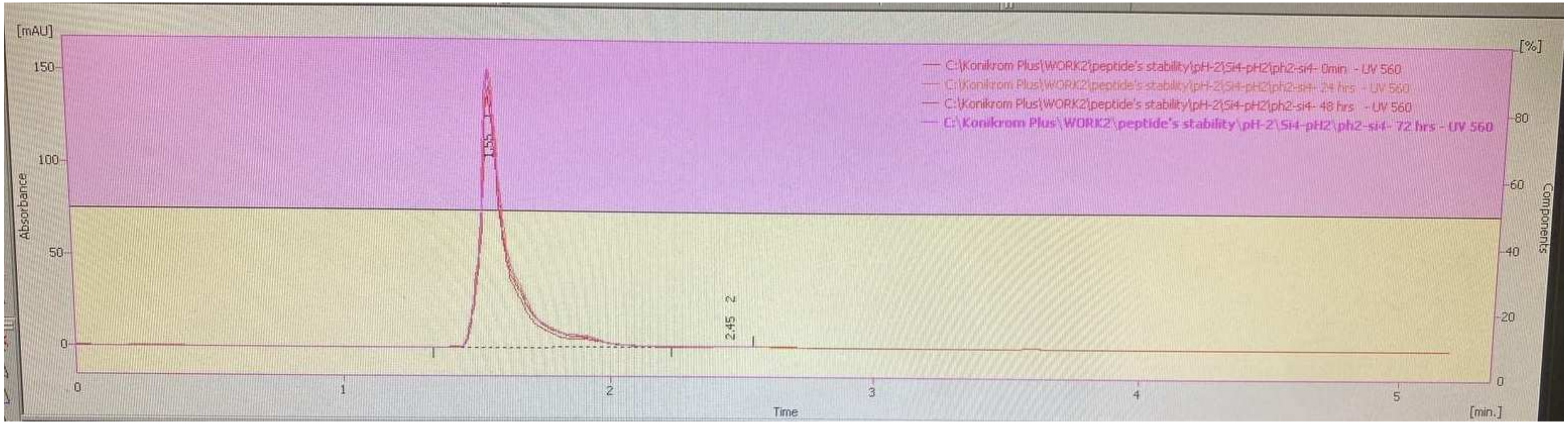

Si5

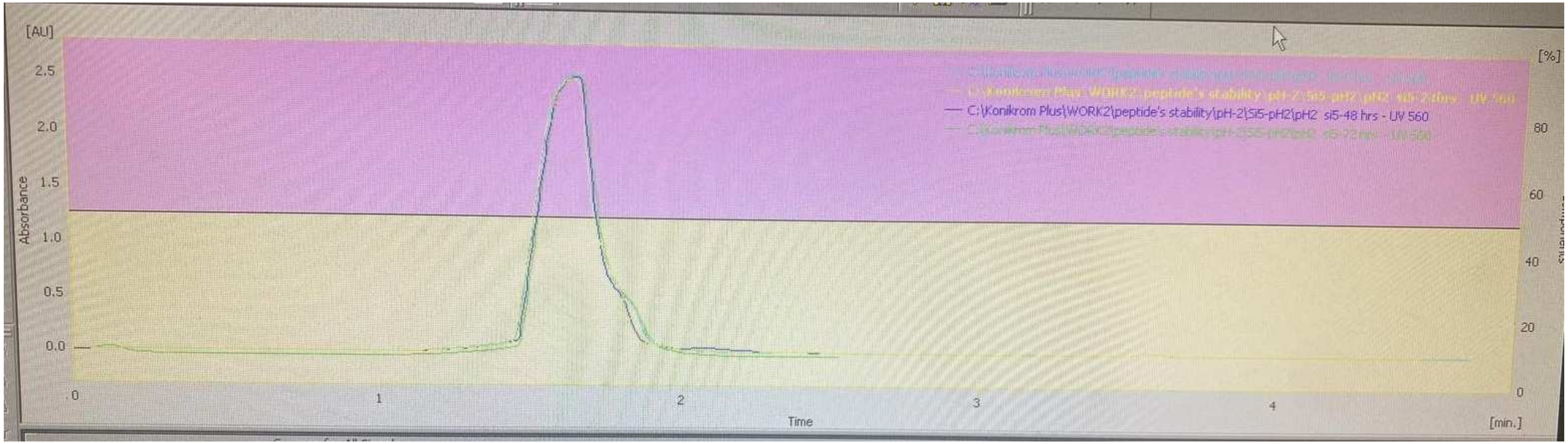

Si9

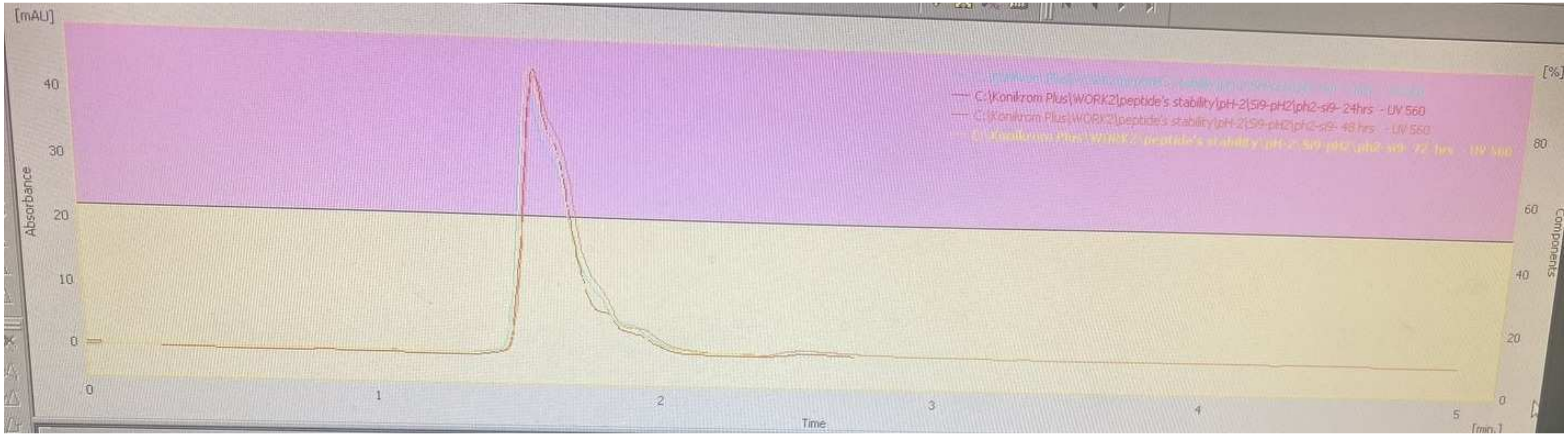
[mAU]
Absorbance
C:\Konikrom Plus\WORK2\peptide's stability\pH-2\Si9-pH2\ph2-si9- 24hrs - UV 560
C:\Konikrom Plus\WORK2\peptide's stability\pH-2\Si9-pH2\ph2-si9- 48 hrs - UV 560
[%]
Components
Time
[min.]

Si10

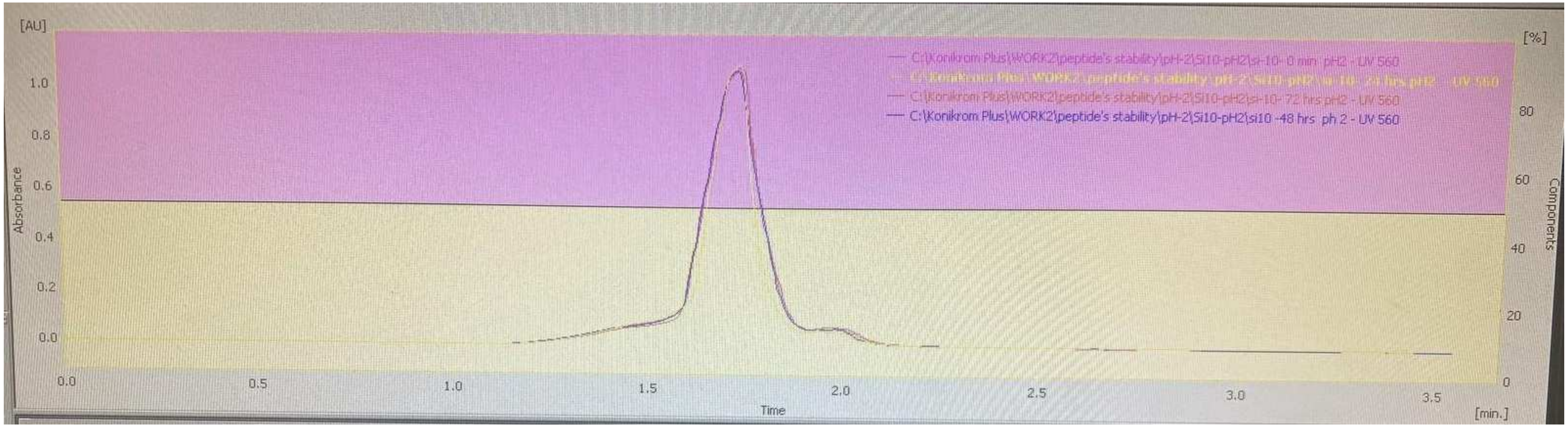
[AU]
Absorbance
C:\Konikrom Plus\WORK2\peptide's stability\pH-2\Si10-pH2\si-10- 0 min pH2 - UV 560
C:\Konikrom Plus\WORK2\peptide's stability\pH-2\Si10-pH2\si-10- 72 hrs pH2 - UV 560
C:\Konikrom Plus\WORK2\peptide's stability\pH-2\Si10-pH2\si10 -48 hrs ph 2 - UV 560
[%]
Components
Time
[min.]

Si12

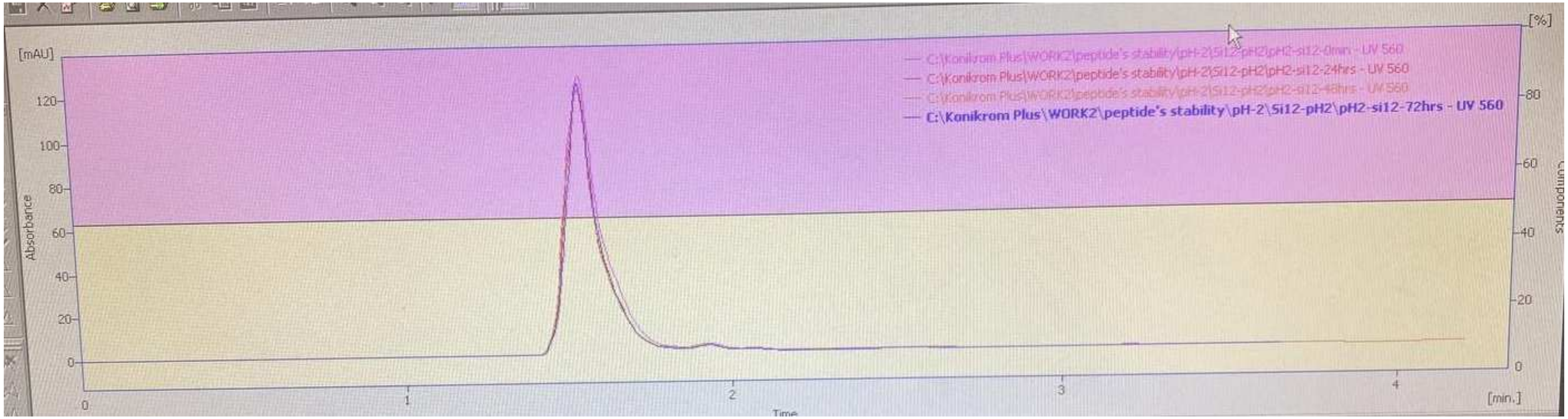
[mAU]
[%]
C:\Konikrom Plus\WORK2\peptide's stability\pH-2\Si12-pH2\pH2-si12-0min - UV 560
C:\Konikrom Plus\WORK2\peptide's stability\pH-2\Si12-pH2\pH2-si12-24hrs - UV 560
C:\Konikrom Plus\WORK2\peptide's stability\pH-2\Si12-pH2\pH2-si12-48hrs - UV 560
C:\Konikrom Plus\WORK2\peptide's stability\pH-2\Si12-pH2\pH2-si12-72hrs - UV 560
Absorbance
Components
120
100
80
60
40
20
0
1
2
3
4
[min.]
Time

pH7.4

Si4

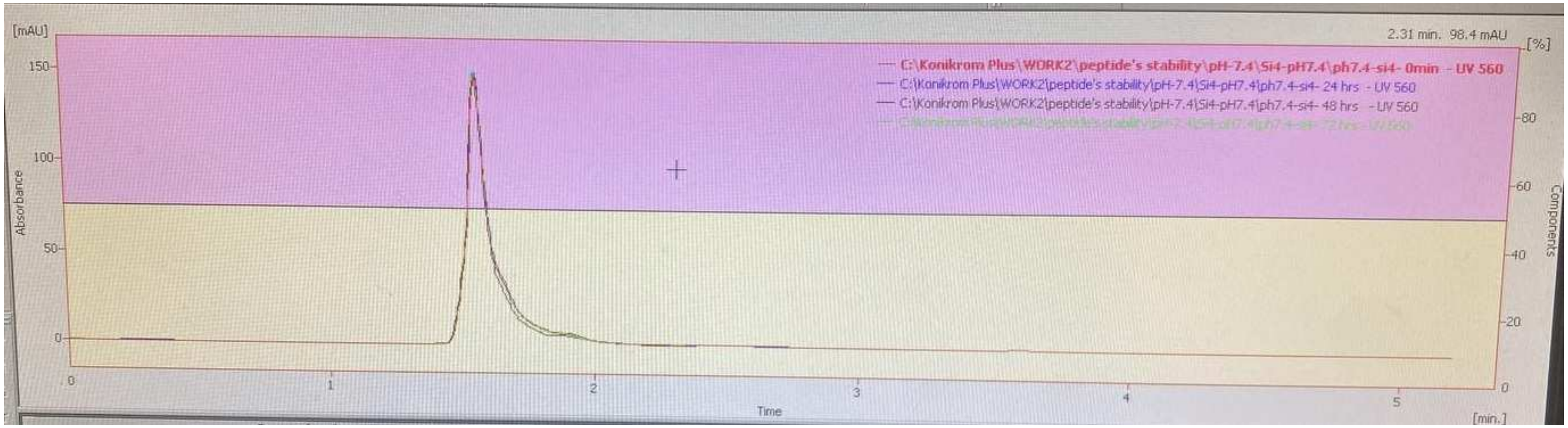
[mAU]
2.31 min. 98.4 mAU
[%]
C:\Konikrom Plus\WORK2\peptide's stability\pH-7.4\Si4-pH7.4\ph7.4-si4- 0min - UV 560
C:\Konikrom Plus\WORK2\peptide's stability\pH-7.4\Si4-pH7.4\ph7.4-si4- 24 hrs - UV 560
C:\Konikrom Plus\WORK2\peptide's stability\pH-7.4\Si4-pH7.4\ph7.4-si4- 48 hrs - UV 560
Absorbance
Components
Time
[min.]

Si5

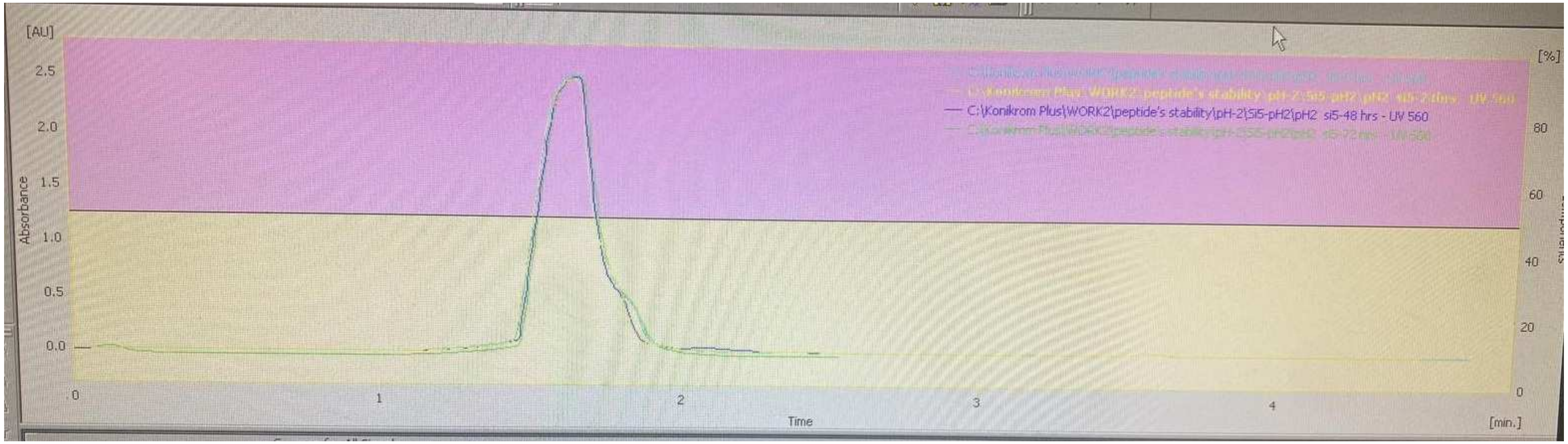
[AU]
[%]
C:\Konikrom Plus\WORK2\peptide's stability\pH-2\Si5-pH2\pH2 si5-48 hrs - UV 560
Absorbance
Time
[min.]

Si9

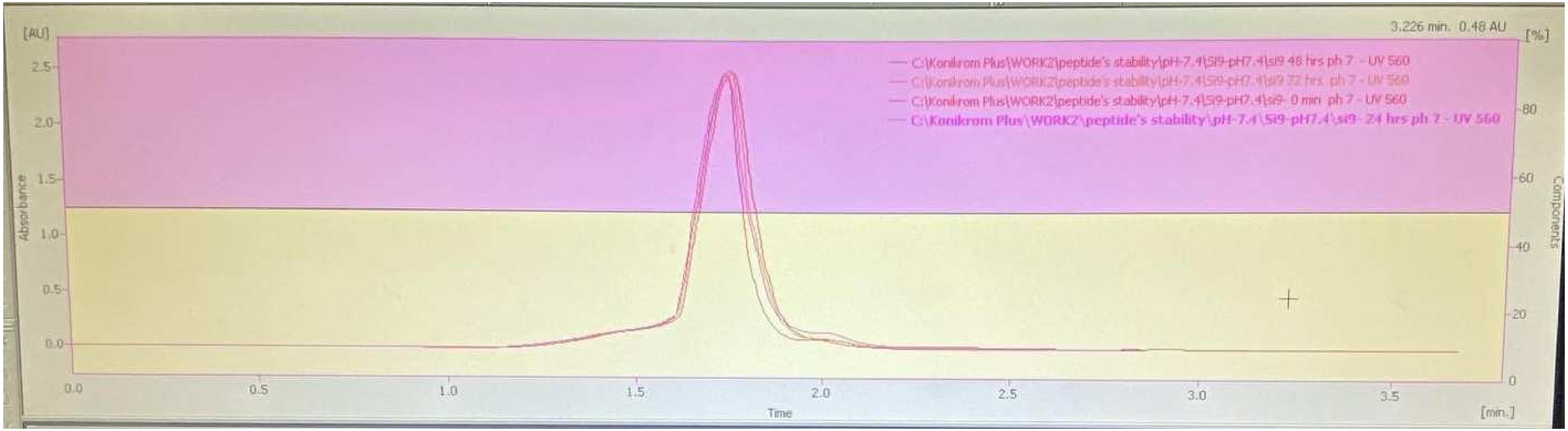

[AU]
3.226 min. 0.48 AU
[%]
C:\Konikrom Plus\WORK2\peptide's stability\pH-7.4\Si9-pH7.4\si9 48 hrs ph 7 - UV 560
C:\Konikrom Plus\WORK2\peptide's stability\pH-7.4\Si9-pH7.4\si9 72 hrs ph 7 - UV 560
C:\Konikrom Plus\WORK2\peptide's stability\pH-7.4\Si9-pH7.4\si9- 0 min ph 7 - UV 560
C:\Konikrom Plus\WORK2\peptide's stability\pH-7.4\Si9-pH7.4\si9- 24 hrs ph 7 - UV 560
Absorbance
Components
Time
[min.]

Si10

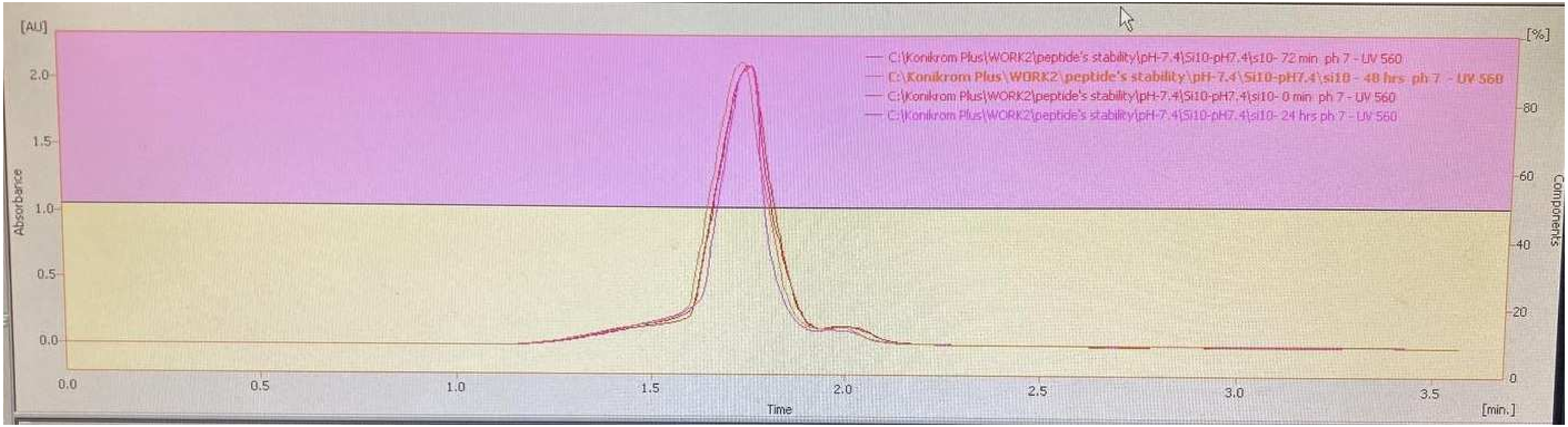

[AU]
[%]
C:\Konikrom Plus\WORK2\peptide's stability\pH-7.4\Si10-pH7.4\si10- 72 min ph 7 - UV 560
C:\Konikrom Plus\WORK2\peptide's stability\pH-7.4\Si10-pH7.4\si10 - 48 hrs ph 7 - UV 560
C:\Konikrom Plus\WORK2\peptide's stability\pH-7.4\Si10-pH7.4\si10- 0 min ph 7 - UV 560
C:\Konikrom Plus\WORK2\peptide's stability\pH-7.4\Si10-pH7.4\si10- 24 hrs ph 7 - UV 560
Absorbance
Components
Time
[min.]

Si12

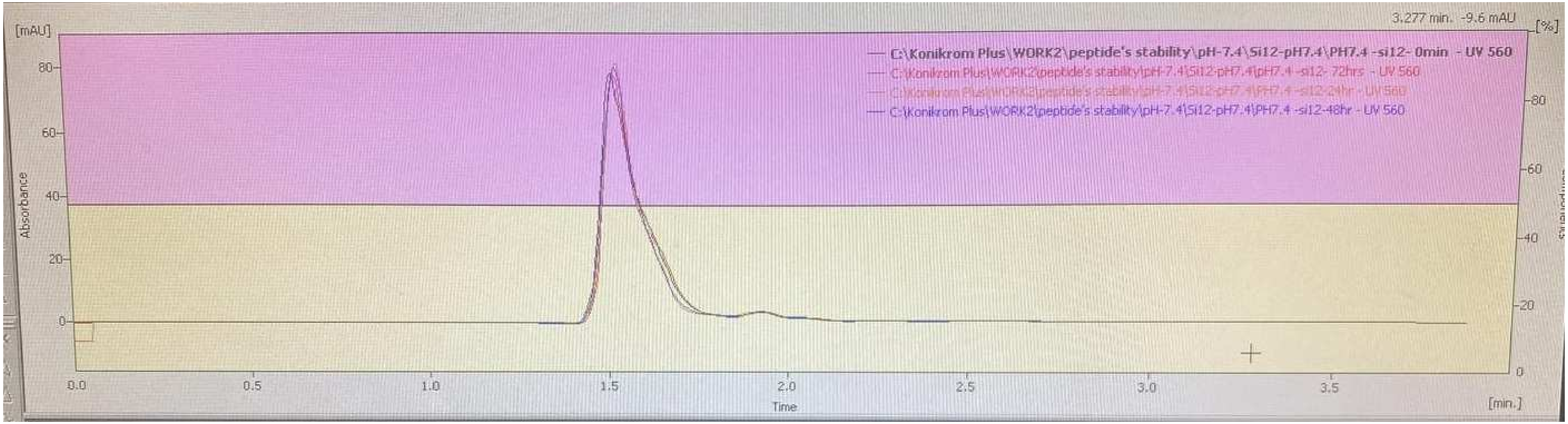
3.277 min. -9.6 mAU
[mAU]
[%]
C:\Konikrom Plus\WORK2\peptide's stability\pH-7.4\Si12-pH7.4\PH7.4 -si12- 0min - UV 560
C:\Konikrom Plus\WORK2\peptide's stability\pH-7.4\Si12-pH7.4\pH7.4 -si12- 72hrs - UV 560
C:\Konikrom Plus\WORK2\peptide's stability\pH-7.4\Si12-pH7.4\PH7.4 -si12-24hr - UV 560
C:\Konikrom Plus\WORK2\peptide's stability\pH-7.4\Si12-pH7.4\PH7.4 -si12-48hr - UV 560
Absorbance
80
60
40
20
0
0.0
0.5
1.0
1.5
2.0
2.5
3.0
3.5
Time
[min.]

pH9

Si4

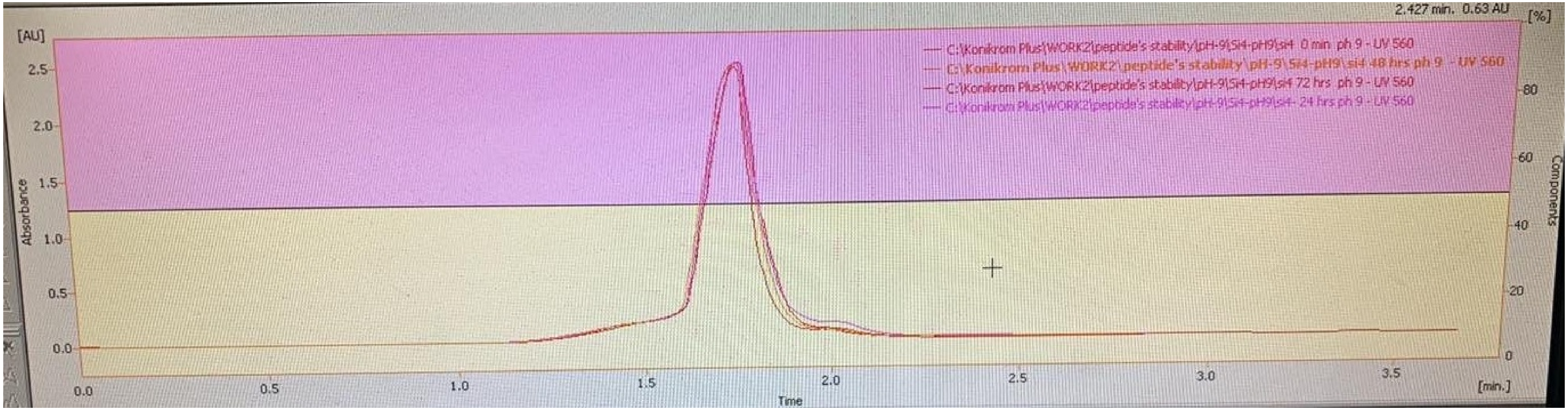
2.427 min. 0.63 AU
[AU]
[%]
C:\Konikrom Plus\WORK2\peptide's stability\pH-9\Si4-pH9\si4 0 min ph 9 - UV 560
C:\Konikrom Plus\WORK2\peptide's stability\pH-9\Si4-pH9\si4 48 hrs ph 9 - UV 560
C:\Konikrom Plus\WORK2\peptide's stability\pH-9\Si4-pH9\si4 72 hrs ph 9 - UV 560
C:\Konikrom Plus\WORK2\peptide's stability\pH-9\Si4-pH9\si4- 24 hrs ph 9 - UV 560
Absorbance
Components
Time
[min.]

Si5

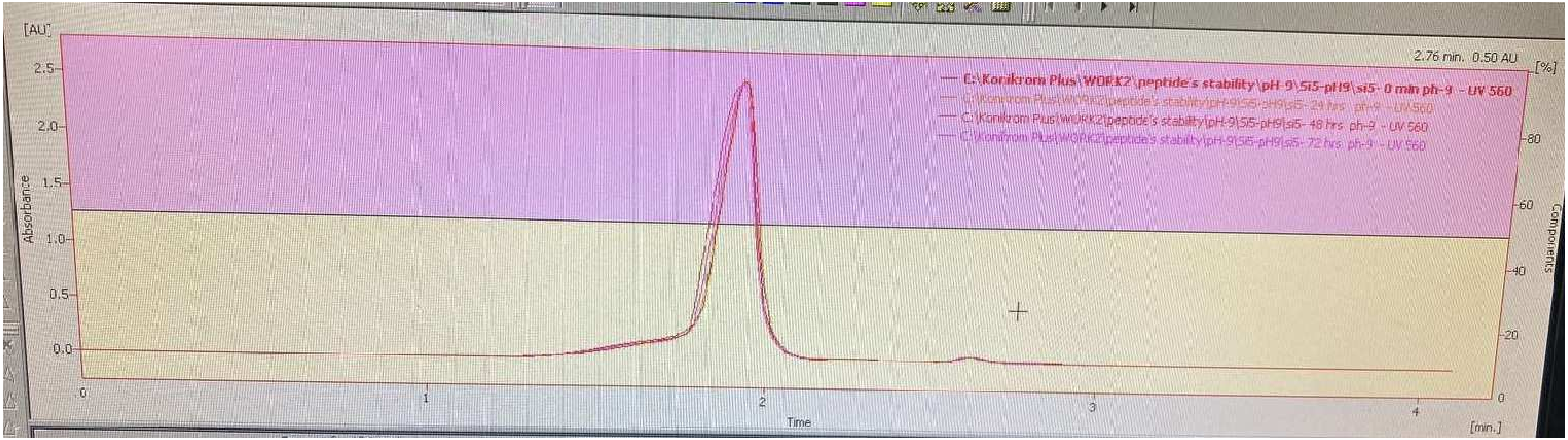
[AU]
2.76 min. 0.50 AU
[%]
C:\Konikrom Plus\WORK2\peptide's stability\pH-9\Si5-pH9\si5- 0 min ph-9 - UV 560
C:\Konikrom Plus\WORK2\peptide's stability\pH-9\Si5-pH9\si5- 48 hrs ph-9 - UV 560
Absorbance
Components
Time
[min.]

Si9

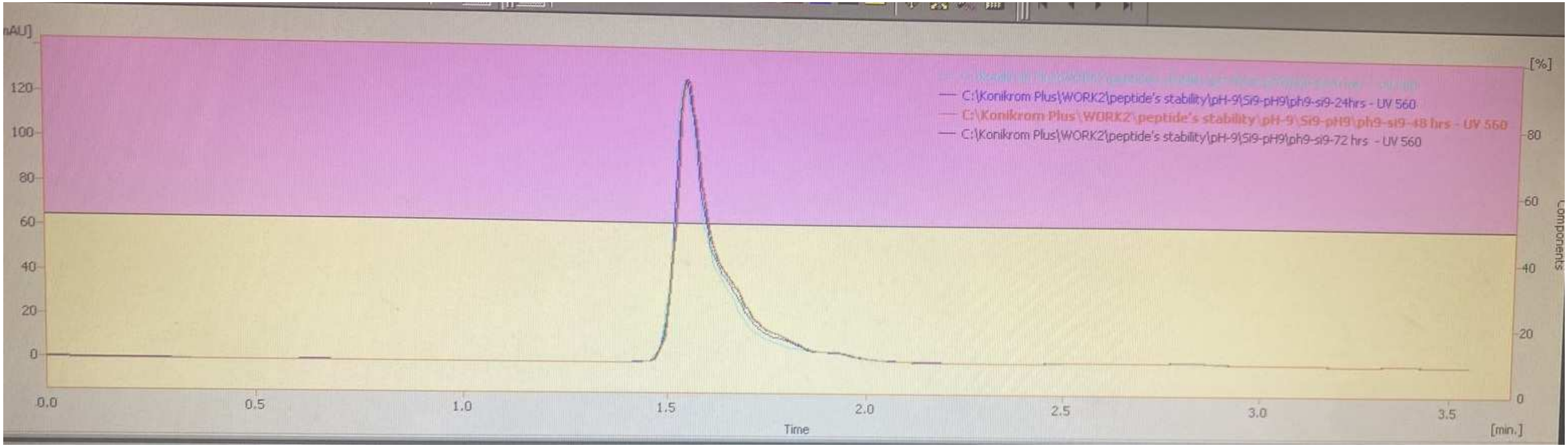
[mAU]
120
100
80
60
40
20
0
[%]
80
60
40
20
0
Components
C:\Konikrom Plus\WORK2\peptide's stability\pH-9\Si9-pH9\ph9-si9-24hrs - UV 560
C:\Konikrom Plus\WORK2\peptide's stability\pH-9\Si9-pH9\ph9-si9-48 hrs - UV 560
C:\Konikrom Plus\WORK2\peptide's stability\pH-9\Si9-pH9\ph9-si9-72 hrs - UV 560
0.0
0.5
1.0
1.5
2.0
2.5
3.0
3.5
Time
[min.]

Si10

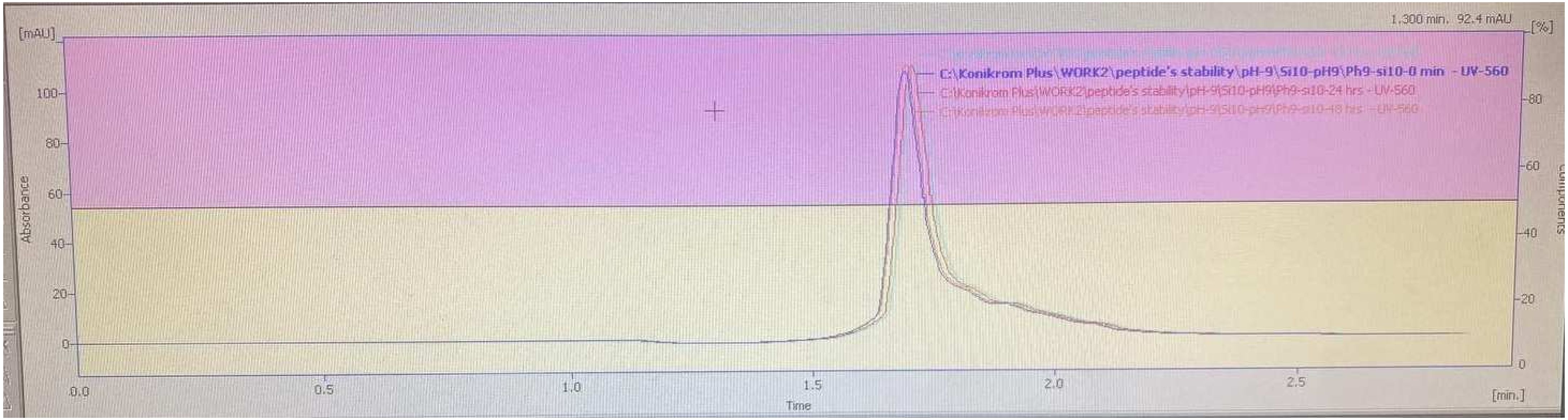
1.300 min. 92.4 mAU
[mAU]
100
80
60
40
20
0
Absorbance
[%]
80
60
40
20
0
Components
C:\Konikrom Plus\WORK2\peptide's stability\pH-9\Si10-pH9\Ph9-si10-0 min - UV-560
C:\Konikrom Plus\WORK2\peptide's stability\pH-9\Si10-pH9\Ph9-si10-24 hrs - UV-560
0.0
0.5
1.0
1.5
2.0
2.5
Time
[min.]

Si12

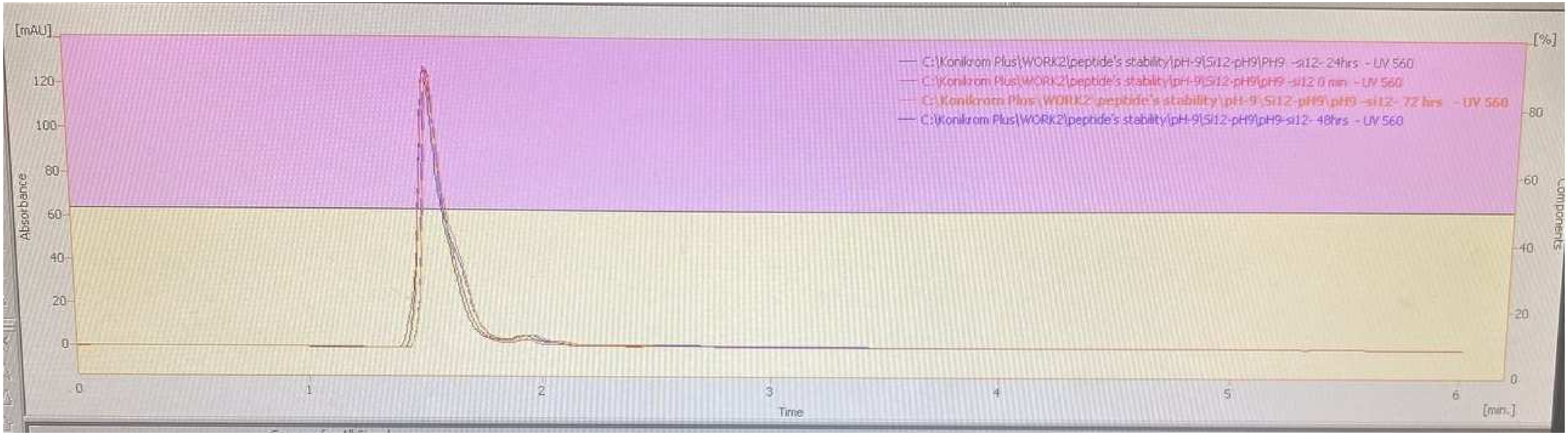

[mAU]
120
100
80
60
40
20
0
Absorbance
C:\Konikrom Plus\WORK2\peptide's stability\pH-9\Si12-pH9\PH9 -si12- 24hrs - UV 560
C:\Konikrom Plus\WORK2\peptide's stability\pH-9\Si12-pH9\pH9 -si12 0 min - UV 560
C:\Konikrom Plus\WORK2\peptide's stability\pH-9\Si12-pH9\pH9 -si12- 72 hrs - UV 560
C:\Konikrom Plus\WORK2\peptide's stability\pH-9\Si12-pH9\pH9-si12- 48hrs - UV 560
[%]
80
60
40
20
0
Components
0
1
2
3
4
5
6
Time
[min.]
